# Heparanase 2 (Hpa2)- a new player essential for pancreatic acinar cell differentiation

**DOI:** 10.1038/s41419-023-05990-y

**Published:** 2023-07-25

**Authors:** Yasmin Kayal, Uri Barash, Inna Naroditsky, Neta Ilan, Israel Vlodavsky

**Affiliations:** 1 Technion Integrated Cancer Center, Rappaport Faculty of Medicine, Technion, Haifa, Israel; 2 Department of Pathology, Rambam Health Care Campus, Haifa, Israel

**Keywords:** Mechanisms of disease, Cancer microenvironment

## Abstract

Heparanase 2 (Hpa2, *HPSE2*) is a close homolog of heparanase. Hpa2, however, lacks intrinsic heparan sulfate (HS)-degrading activity, the hallmark of heparanase enzymatic activity. Mutations of HPSE2 were identified in patients diagnosed with urofacial syndrome (UFS), a rare genetic disorder that exhibits abnormal facial expression and bladder voiding dysfunction, leading to renal damage and eventually renal failure. In order to reveal the role of HPSE2 in tissue homeostasis, we established a conditional Hpa2-KO mouse. Interestingly, the lack of Hpa2 was associated with a marked decrease in the expression of key pancreatic transcription factors such as PTF1, GATA6, and Mist1. This was associated with a two-fold decrease in pancreas weight, increased pancreatic inflammation, and profound morphological alterations of the pancreas. These include massive accumulation of fat cells, possibly a result of acinar-to-adipocyte transdifferentiation (AAT), as well as acinar-to-ductal metaplasia (ADM), both considered to be pro-tumorigenic. Furthermore, exposing Hpa2-KO but not wild-type mice to a carcinogen (AOM) and pancreatic inflammation (cerulein) resulted in the formation of pancreatic intraepithelial neoplasia (PanIN), lesions that are considered to be precursors of invasive ductal adenocarcinoma of the pancreas (PDAC). These results strongly support the notion that Hpa2 functions as a tumor suppressor. Moreover, Hpa2 is shown here for the first time to play a critical role in the exocrine aspect of the pancreas.

## Introduction

Activity capable of cleaving macromolecular heparin at a limited number of sites was first reported already in 1975 [1]. Endoglycosidase activity that degrades heparan sulfate (HS)-polymers into oligosaccharides was reported soon after [2] and the activity responsible for HS-cleavage was termed heparanase. Given the structural role of HS proteoglycans (HSPG) in the assembly of extracellular matrix (ECM) and basement membrane, it was hypothesized that HS-degrading activity (heparanase) will result in remodeling of the ECM, most likely associating with sprouting of new blood vessels (angiogenesis) and cell dissemination accompanying with tumor metastasis and transmigration of immune cells [3–5]. Intensive research effort in the last two decades conclusively showed that indeed heparanase exerts a strong pro-tumorigenic properties, thus turning heparanase to a valid target for the development of anti-cancer drugs, some of which are under clinical evaluation [6–9].

HPSE2, the gene encoding heparanase 2 (Hpa2), was cloned soon after the cloning of heparanase, based on sequence homology [10]. Interestingly, Hpa2 lacks intrinsic HS-degrading activity, the hallmark of heparanase [11], yet retains the capacity to interact with HS [11]. In fact, Hpa2 exhibits an even higher affinity towards heparin and HS than heparanase [11], thus competing for HS binding and thereby inhibiting heparanase enzymatic activity [11]. Unlike the intense research effort devoted to exploring the significance of heparanase in cancer progression, very little attention was given to Hpa2. Evidence gathered in recent years suggests, nonetheless, that Hpa2 functions to attenuate tumor growth. Clinically, it was reported that, unlike heparanase, Hpa2 expression is readily detected in normal epithelium of the bladder, breast, cervical, gastric, and ovarian tissues, whereas its expression is substantially decreased in the resulting carcinomas [12–17], expression pattern that is typical of a tumor suppressor. Furthermore, patients that retain high levels of Hpa2 survived longer than patients bearing Hpa2-low tumors [11, 15, 16, 18, 19]. Experimentally, overexpression of Hpa2 attenuated the growth of tumor xenografts, whereas Hpa2 gene silencing resulted in bigger tumors [13, 15, 16, 18, 20–22]. This is best demonstrated by gene editing of Hpa2 in pharyngeal FaDu cells applying the CRISPR technology. Notably, Hpa2-null cells produced bigger tumors vs control cells, whereas the rescue of Hpa2 in the null cells resulted in smaller tumors [21], supporting the notion that Hpa2 functions as a tumor suppressor. Recently, we reported that these features are also evident in pancreatic carcinoma [18], a most aggressive type of cancer. In this and other studies, we focused on the role of Hpa2 in the tumor cells and the consequences of Hpa2 overexpression or gene silencing on tumor growth [13, 15, 16, 18, 20–22]. However, the role of host Hpa2 in tumorigenesis was not explored yet. To examine this aspect we generated a Hpa2-knockout (KO) mouse. In this conditional mouse model, tamoxifen-inducible Cre-mediated recombination results in the removal of exon 5 and the disruption of HPSE2 open reading frame in essentially all cells and tissues. Intriguingly, the lack of Hpa2 was associated with increased pancreatic inflammation and profound morphological alterations of the pancreas. These include massive accumulation of fat cells, possibly a result of acinar-to-adipocyte transdifferentiation (AAT), as well as acinar-to-ductal metaplasia (ADM), both considered to be pro-tumorigenic. Indeed, once implanted orthotopically, Panc-02 mouse pancreatic carcinoma cells developed 3-fold bigger tumors in Hpa2-KO vs control wild type (wt) pancreas. Furthermore, exposing mice to a carcinogen (AOM) and pancreatic inflammation (cerulein) resulted in the formation of pancreatic intraepithelial neoplasia (PanIN), lesions that are considered to be precursors of invasive ductal adenocarcinoma of the pancreas (PDAC), only in the Hpa2-KO pancreas. These results strongly support the notion that Hpa2 functions as a tumor suppressor. Moreover, Hpa2 is shown here for the first time to play a critical role in the exocrine aspect of the pancreas, modulating the expression of key transcription factors such as PTF1, GATA6, and Mist1.

## Results

### Hpa2 deficiency results in fatty pancreas and ADM

**Fig. 1:**
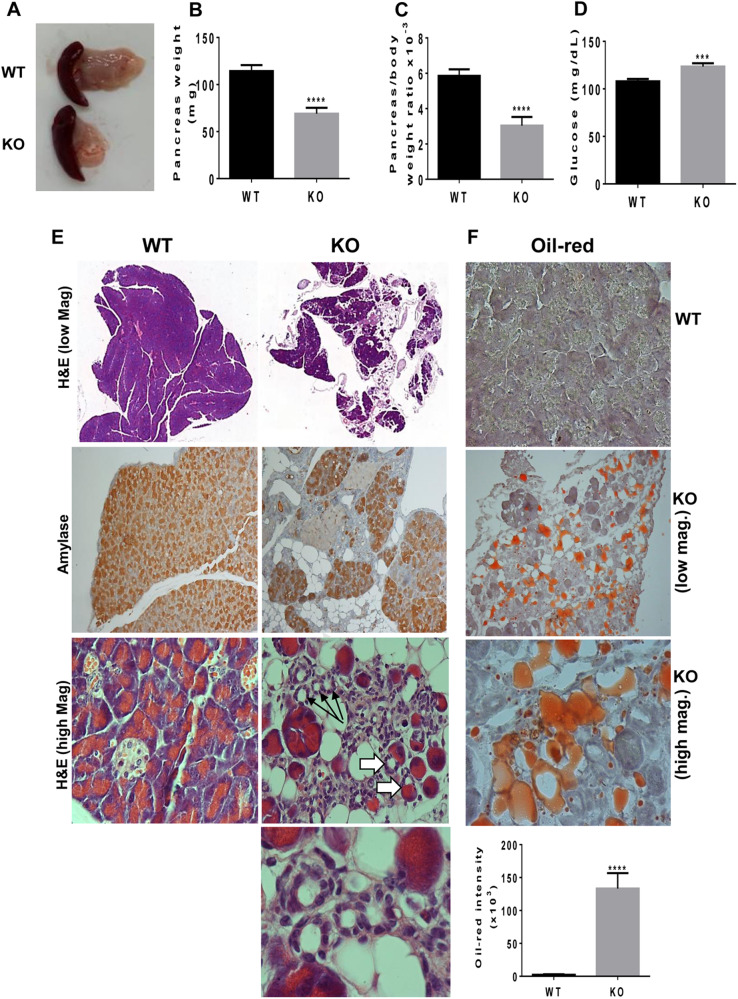
Hpa2-KO pancreas is smaller and exhibits abnormal morphology. **A** Pancreas weight. Pancreas tissue was harvested from *wt* (*n* = 19) and Hpa2-KO mice (*n* = 13), photographed (while still attached to the spleen), and weighed (**B**). Pancreas weight was also calculated relative to body weight (**C**). **D**. Blood glucose was measured in *wt* (*n* = 26) and Hpa2-KO (*n* = 27) mice. **E** Histological analyses. Pancreas was collected from *wt* (*n* = 19) and Hpa2-KO (*n* = 22) female mice and 5-micron sections were subjected to H&E staining shown at low (upper panels) and high (third panels) magnifications. Sections were also subjected to immunostaining with anti-amylase antibody (second panels). Shown are representative images at original magnifications of x10 (upper panel) and x 100 (second and third panels). Arrows point to ADM (third panel; KO); ADM is shown also in the lower right panel at high (×250) magnification. **F** Oil red staining. 5-micron sections of *wt* and Hpa2-KO pancreata (*n* = 7) were subjected to oil red staining essentially as described under 'Materials and Methods’. Shown are representative photomicrographs of oil red stained *wt* (upper panel) and Hpa2-KO pancreata at low (×25; second panel) and high (×100; third panel) magnifications. Quantification of the oil red staining intensity is shown graphically in the lower panel (2.5 ± 0.7 vs 133.4 ± 23.3 × 10^3^ pixels/high power field for *wt* and Hpa2-KO pancreas, respectively; *p* < 0.0001).

**Fig. 2:**
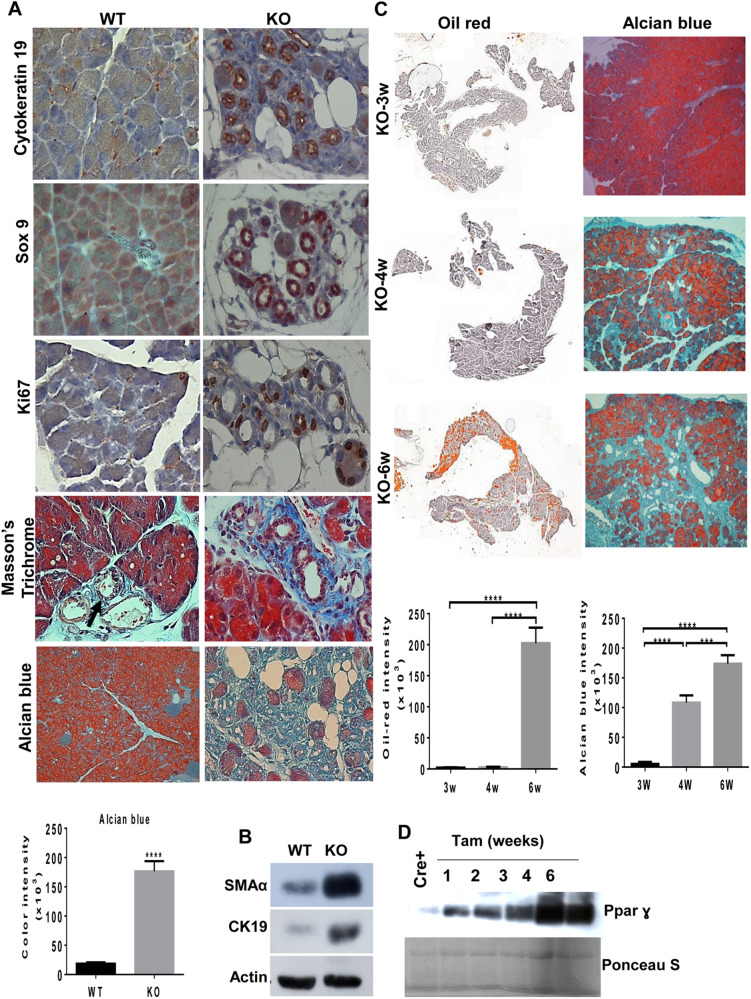
Hpa2-KO pancreas undergoes ADM. **A** Immunostaining. 5-micron sections of *wt* and Hpa2 pancreas (*n* = 7) were subjected to immunostaining applying anti-cytokeratin 19 (upper panels), anti-Sox 9 (second panels), and anti-Ki67 (third panels) antibodies. Shown are representative images at original magnifications of x100. Sections were also subjected to Masson’s Trichrome staining (fourth panels) which stains collagen fibers blue, and alcian blue (lower panels) which stains HS blue. Quantification of the alcian blue staining is shown graphically in the lower panel. **B** Immunoblotting. Extracts of *wt* and Hpa2-KO pancreas tissue were subjected to immunoblotting applying anti-SMAα (upper panel), anti-cytokeratin 19 (CK19; second panel), and anti-actin (lower panel) antibodies. **C** Kinetics. Pancreas tissues were collected from Cre+ mice (*n* = 7) and at 3 (upper), 4 (second), and 6 (third panel) weeks (*n* = 9) following the administration of tamoxifen and 5-micron sections were subjected to oil-red staining. Quantification of the oil red staining intensities is shown graphically in the lower panel. **D** Induction of Pparɣ. Pancreas tissues were collected from Cre+ mice before (Cre+) and 1, 2, 3, 4, and 6 weeks after the administration of tamoxifen (Tam). Pancreas extracts (*n* = 7) were then subjected to immunoblotting applying anti- Pparɣ antibody. Ponceau S staining of the membrane is shown in the lower panel.

To reveal the role of host Hpa2 in tumorigenesis we generated a conditional Hpa2-KO mouse. In this mouse strain, exon 5 of HPSE2 is excised by tamoxifen-induced Cre recombination (Suppl. Fig. 1A). We used a transgenic mouse in which expression of the Cre recombinase is driven by the chicken β-actin promoter, thus directing high levels of expression in essentially all cells and tissues. This approach was preferred given the lethality of HPSE2-mutant mice at ~4 weeks of age [23]. We first confirmed that Hpa2 expression is reduced substantially in selected tissues of the newly generated Hpa2-KO mice, including the bladder, stomach, testis, lungs, and pancreas (Suppl. Fig. 1B). Examination of selected organs (i.e., liver, kidney, lungs, and mammary gland) in terms of tissue weight and histology did not reveal overt differences between *wt* and Hpa2-KO mice (not shown). In striking contrast, we observed that the pancreas of Hpa2-KO female mice is smaller (Fig. 1A, B), presenting half the weight of *wt* pancreas when calculated relative to body weight (Fig. 1C; 5.9 ± 0.4 mg/g body weight vs 3 ± 0.2 mg/g for *wt* vs Hpa2-KO pancreas/body weight; *p* < 0.0001). Blood glucose, nonetheless, exhibited normal values in *wt* (~108 ± 2.5 mg/dL) and in Hpa2-KO female (123.4 ± 3.5; Fig. 1D) mice, suggesting that beta-islets are preserved. A closer histological examination revealed significant morphological abnormalities in Hpa2-KO vs *wt* mice. A large proportion (~30%) of the Hpa2-KO pancreas appeared to be fat cells (Fig. 1E, upper panels), replacing the pancreas acinar cells, possibly the result of acinar-to-adipocytes transdifferentiation (AAT) [24]. Thus, while staining for amylase that labels acinar cells, appeared uniform in *wt* pancreas, a mosaic pattern of amylase staining was evident in Hpa2-KO pancreas, in which patches of amylase-positive acinar cell clusters are surrounded by fat cells (Fig. 1E, second panels). Oil red staining further showed a remarkable increase of fat cells accumulating in the Hpa2-KO vs *wt* pancreas (Fig. 1F). Quantification of the staining revealed a marked increase in oil red staining intensity in Hpa2-KO pancreas, exhibiting statistically highly significant differences between *wt* and Hpa2-KO pancreas (Fig. 1F, lower panel; 2.5 ± 0.7 vs 133.4 ± 23.3 for *wt* and Hpa2-KO pancreas, respectively; *p* < 0.0001). In addition, a substantial number of duct-like structures were observed only within the Hpa2-KO pancreas (Fig. 1E, third and lower right panels, arrows). These were stained positive for cytokeratin 19 (Fig. 2A, upper panels) and Sox 9 (Fig. 2A, second panels), and exhibited high proliferative capacity, indicated by a prominent increase of Ki67-positive cells (Fig. 2A, third panels). In addition, these structures deposited large amounts of collagen (Masson’s Trichrome blue staining; Fig. 2A, fourth panels). These characteristics were also evident by immunoblotting for alpha-smooth muscle actin and cytokeratin 19, typical of acinar-to-ductal metaplasia (Fig. 2B), revealing 8- and 4-fold increases as quantified by densitometry analysis, respectively.

Interestingly, ADM structures were stained strongly for alcian blue that labels HS (Fig. 2A, fifth panels). We further used alcian blue staining to quantify the ADM aspect of the Hpa2-KO pancreas. Notably, a 10-fold increase in alcian blue staining intensity was quantified in Hpa2-KO vs *wt* pancreata (Fig. 2A, lower left panel). Kinetic analyses revealed that accumulation of fat cells (stained positive for oil red O) within the pancreas (Fig. 2C, left panels; 2.3 ± 0.4, 2.6 ± 0.7, and 202.4 ± 25 × 10^3^ pixels/high power field for 3 weeks, 4 weeks, and 6 weeks post tamoxifen, respectively; *p* < 0.0001) and ADM (Fig. 2C, right panels; 5.5 ± 3.3, 115.6 ± 10.3, and 173.9 ± 14 × 10^3^ pixels/high power field for 3 weeks, 4 weeks, and 6 weeks post tamoxifen, respectively; *p* < 0.0001, Alcian blue; and Suppl. Fig. 2, H&E) are evident and highly abundant already 4–6 weeks after the administration of tamoxifen, coinciding with the induction of peroxisome proliferator-activated receptor gamma (Ppar ɣ; Fig. 2D) and cytokeratin 19 (CK19; Suppl. Fig. 2, lower right panel), a master regulator of adipogenesis [25] and a marker of ADM, respectively. Likewise, expression of Plin1 and Plin4 that are regulated by Pparɣ and signify adipocytes [26] was induced (3.3- and 1.8-folds, respectively; *p* < 0.05) in Hpa2-KO vs *wt* (Suppl. Fig. 3A) mice, further supporting the fatty nature of Hpa2-deficient pancreas. Altogether, these features signify that Hpa2-KO pancreas undergoes acinar-to-ductal metaplasia (ADM) [27] and turns into a fatty tissue.

### Pancreas of Hpa2-KO male mice is inflamed and is far more sensitive to cerulein

**Fig. 3:**
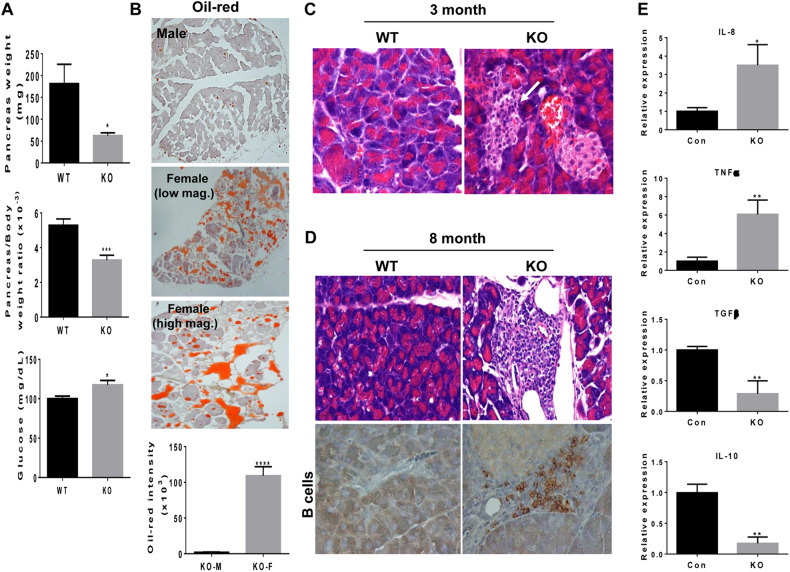
The pancreas of male Hpa2-KO mice is inflamed. **A** Pancreas weight. Pancreata were collected from *wt* (*n* = 9) and Hpa2-KO mice (*n* = 10) and weighed (upper panel); Pancreata weight relative to body weight is presented in the second panel. Blood glucose was measured in *wt* (*n* = 7) and Hpa2-KO (*n* = 8) mice (lower panel). **B**. Oil-red staining. 5-micron sections of *wt* and Hpa2-KO pancreas were subjected to oil-red staining as described under 'Materials and Methods'. Shown are representative images at low (upper and second panels) and high (third panel) magnifications. Quantification of the oil-red staining intensities is presented graphically in the lower panel (2.02 ± 0.4 vs 109.2 ± 12.7 × 10^3^ pixels/high power field for male (m) and female (f) Hpa2-KO pancreas, respectively; *p* < 0.0001). **C, D**. Histological analyses. 5-micron sections of 3-months-old and 8-months-old *wt* and Hpa2-KO pancreas were subjected to H&E staining. 8-months old sections were subjected to immunostaining applying anti CD45R that specifically labels B cells. Original magnifications: ×100. White arrow in (**C**) points to inflammatoty foci next to a beta islet. **E** Total RNA was extracted from 3-months old *wt* and Hpa2-KO pancreas (*n* = 5) and subjected to qPCR analyses applying primer sets specific for the indicated cytokines.

Similar analyses of Hpa2-KO male pancreas revealed some differences. As described above for females, the weight of Hpa2-KO male pancreas was reduced nearly 2-fold vs *wt* pancreas (5.3 ± 0.4 mg/g body weight vs 3.3 ± 0.3 mg/g for *wt* vs Hpa2-KO pancreas/body weight; Fig. 3A; *p* = 0.0005), yet exhibited normal blood glucose levels (Fig. 3A, lower panel). However, unlike female Hpa2-KO mice, the male Hpa2-KO pancreas did not exhibit accumulation of fat (Fig. 3B; 2.02 ± 0.4 vs 109.2 ± 12.7 × 10^3^ pixels per high power field for male vs female Hpa2-KO mice, respectively; *p* < 0.0001). Closer histological examination did not reveal evidence of AAT or ADM. However, foci of inflammation were readily detected within the Hpa2-KO pancreas of young (3-month-old; Fig. 3C, white arrow) and older (8-month-old; Fig. 3D, upper panels) male mice. Pathological evaluation characterized these foci as acute (3-month old; Fig. 3C) and chronic (8-month old; Fig. 3D, upper panels) inflammation. This was further confirmed by positive staining of 8-month old male Hpa2-KO pancreas for B cells (Fig. 3D, lower panels). Applying pancreas extracts onto antibody array further revealed a substantial increase in the expression levels of many cytokines by Hpa2-KO male pancreas (Suppl. Fig. 3B), and increased expression of selected cytokines (IL-8, TNFα) was further confirmed by qPCR (Fig. 3E, upper panels). In addition, expression of anti-tumorigenic TGFβ and IL-10 was reduced in the Hpa2-KO pancreas (Fig. 3E, lower panels), thus establishing a pro-tumorigenic niche.

**Fig. 4:**
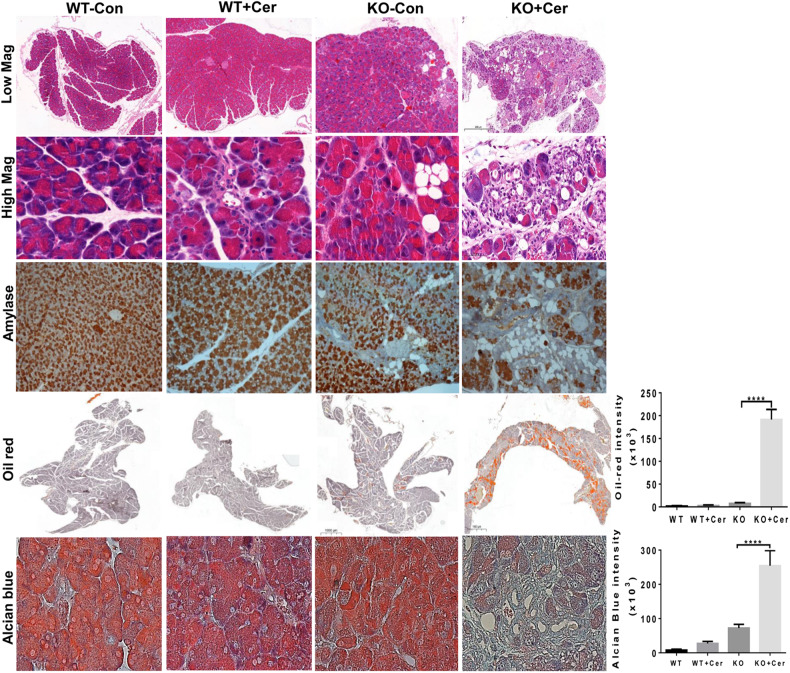
Male Hpa2-KO mice respond vigorously to cerulein. *wt* and Hpa2-KO male mice were administrated with saline (Con) or cerulein (50 µg/kg; 6 hourly injections). Mice (7 per group) were sacrificed 24 h later, pancreata were collected, fixed in formalin and embedded in paraffin. 5-micron sections were subjected to H&E (upper and second panels) and oil-red (fourth panels) staining. Sections were also subjected to immunostaining applying anti-amylase antibodies (third panels). Shown are representative images at low (×5; upper and fourth panels) and high (×100; second, third and fifth panels) magnifications. Quantification of the oil-red staining intensities is presented graphically in the fourth right panel. Sections were similarly stained with alcian blue (fifth panels). Quantification of the alcian blue staining intensities is presented graphically in the lower right panel. Note the massive accumulation of fat cells and ADM only in Hpa2-KO mice following cerulein treatment.

To examine the response of Hpa2-KO mice to cerulein, best recognized for its capacity to induce acute pancreatitis, we next utilized male mice in which the pancreas morphology is relatively preserved. Mice were administrated with 6 hourly injections of saline (Con) or cerulein (50 µg/kg, i.p) and were sacrificed 24 h later. In general, *wt* C57BL/6 mice responded poorly to cerulein (Fig. 4, left panels). In striking contrast, cerulein profoundly affected the pancreas of Hpa2-KO mice (Fig. 4, right panels). Histological examination revealed abundance of fat cells in cerulein-treated Hpa2-KO pancreas, evidenced by H&E (Fig. 4, upper and second right panels), amylase (Fig. 4, third right panels), and oil red (Fig. 4, fourth right panels) staining, altogether clearly displaying a remarkable decrease in the number of amylase-positive acinar cells and accumulation of fat cells in male Hpa2-KO pancreas following cerulein treatment. Quantification of the oil red staining revealed an extensive increase in oil red staining in Hpa2-KO pancreas following cerulein treatment (Fig. 4, lower right panel; 1.8 ± 0.2, 2.8 ± 0/4, 7.7 ± 1.7, and 191.6 ± 21.9 × 10^3^ pixels/high power field for *wt*, *wt* + Cer, KO, KO+Cer pancreas, respectively; *p* < 0.0001). Cerulein also elicited ADM in Hpa2-KO, but not *wt*, pancreas as indicated by alcian blue staining (Fig. 4, lower panels; *p* < 0.001), cytokeratin 19 and Sox9 immunostaining (Suppl. Fig. 4, upper and second panels), and the recruitment of immune cells, mostly macrophages, indicated by immunostaining for F4/80 (Suppl. Fig. 4, lower right panels). Thus, within one day, cerulein treatment converted the relatively preserved morphology of Hpa2-KO male pancreas to the morphology that is typical for untreated female Hpa2-KO pancreas. This implies that although the male pancreas appeared relatively normal in terms of morphology, abnormal cellular and molecular mechanism(s) were already turned on. This suggests that Hpa2 functions to protect the pancreas against inflammation and pancreatitis.

### Impaired pancreas morphology in Hpa2-KO mice is not reversed by heparanase inhibitors

**Fig. 5:**
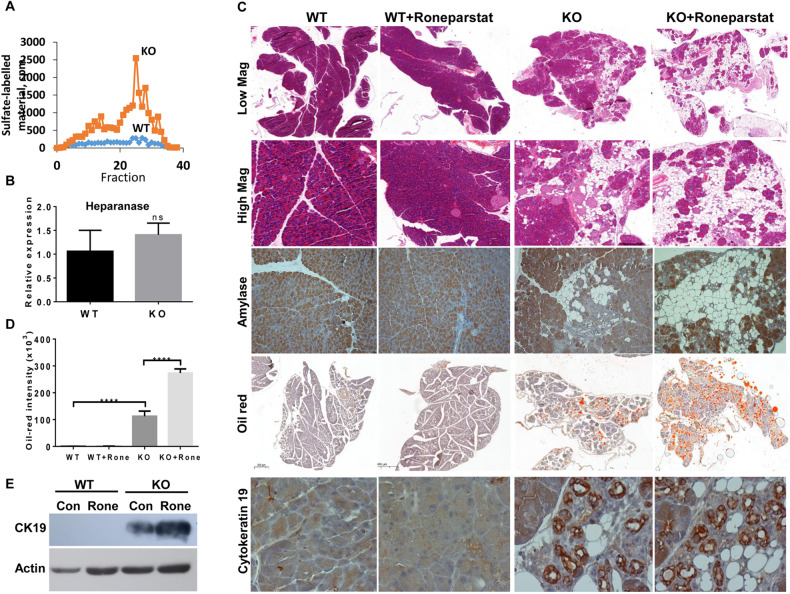
Heparanase inhibitors do not reverse the morphology of Hpa2-KO pancreas. **A** Heparanase enzymatic activity. Pancreas tissue was collected from *wt* and Hpa2-KO female mice (*n* = 5), homogenized, applied onto dishes coated with ^35^S-labelled ECM, and heparanase activity was determined as described under ‘Materials and Methods’. Note increased heparanase activity in Hpa2-KO pancreas. **B** Total RNA was extracted from another portion of the pancreas and subjected to qPCR applying primers specific for heparanase. Heparanase expression in Hpa2-KO pancreas is presented relative to *wt* pancreas, set arbitrarily to a value of 1, and calculated after normalization to actin. **C** Histological evaluation. *wt* (*n* = 15) and Hpa2-KO mice (*n* = 12) were administrated with PBS (Con) or Roneparstat (1500 µg/mouse, twice daily) for 10 days. At termination, pancreata were collected, small portion was used for protein and RNA extractions and the rest of the tissue was fixed in formalin and embedded in paraffin. 5-micron sections were subjected to H&E staining (upper and second panels) or were subjected to immunostaining applying anti-amylase (third panels) and anti-cytokeratin 19 (lower panels) antibodies. Frozen sections were similarly prepared (*n* = 7) and subjected to oil red staining, essentially as described under ‘Materials and Methods’ (fourth panels). Quantification of the oil red staining is shown graphically in (**D**). **E**. Immunoblotting. Pancreata extracts were subjected to immunoblotting applying anti-cytokeratin 19 (CK19; upper panel) and anti-actin (lower panel) antibodies.

We have reported previously that Hpa2 can inhibit heparanase enzymatic activity [11], leading to the concept that Hpa2 is a natural inhibitor of heparanase. Thus, the heparanase:Hpa2 ratio is thought to play an important role in human pathologies [28–30]. In line with this notion, we found that heparanase activity is increased markedly in female Hpa2-KO vs *wt* pancreas (Fig. 5A) whereas heparanase expression is unchanged (Fig. 5B). Similar increase in heparanase activity was measured in Hpa2-KO male pancreas (Suppl. Fig. 3C). We, therefore, suspected that the impaired morphology of female Hpa2-KO pancreas is due to the high levels of heparanase activity. To examine this possibility we treated *wt* and Hpa2-KO female mice with the heparanase inhibitor Roneparstat (SST0001) which was evaluated clinically in cancer patients [7]. Roneparstat did not appear to affect the *wt* pancreas (Fig. 5C, left panels). In striking contrast, Roneparstat profoundly affected Hpa2-KO pancreas (Fig. 5C, right panels). Unexpectedly, rather than reversing the pancreas morphology, Roneparstat promoted the morphological abnormalities that characterize female Hpa2-KO pancreas, resulting in more fat cells (Fig. 5C, upper and second right panels) and consequently less amylase-positive acinar cells (Fig. 5C, third right panels), also evident by increased oil red staining (Fig. 5C, fourth panels). Quantification of the oil red staining revealed a 2.4-fold increase of fat content in Roneparstat-treated vs untreated female Hpa2-KO pancreas (Fig. 5D; 0.6 ± 0.2, 0.6 ± 0.4, 112.5 ± 19.4, 273.4 ± 15.3 for *wt, wt*+Rone, KO, KO+Rone × 10^3^ pixels per high power field, respectively; *p* < 0.0001). Moreover, ADM was also promoted by Roneparstat treatment (Fig. 5C, lower right panels), and this was further confirmed by immunoblotting (Fig. 5E), revealing a 3.3-fold increase in cytokeratin 19 expression as quantified by densitometry analyses. Similar aggravation of Hpa2-KO pancreas morphology was observed by treating Hpa2-KO female mice with another heparanase inhibitor, Pixatimod (PG545) (Suppl. Fig. 5). These results may suggest that heparanase can compensate, to some extent, the absence of Hpa2; lack of both Hpa2 (KO) and heparanase activity (Roneparstat/Pixatimod), is most devastating to the exocrine aspect of the pancreas, resulting in a more severe fatty pancreas and ADM.

### Hpa2 deficiency is associated with decreased expression of GATA6 and PTF1α

**Fig. 6:**
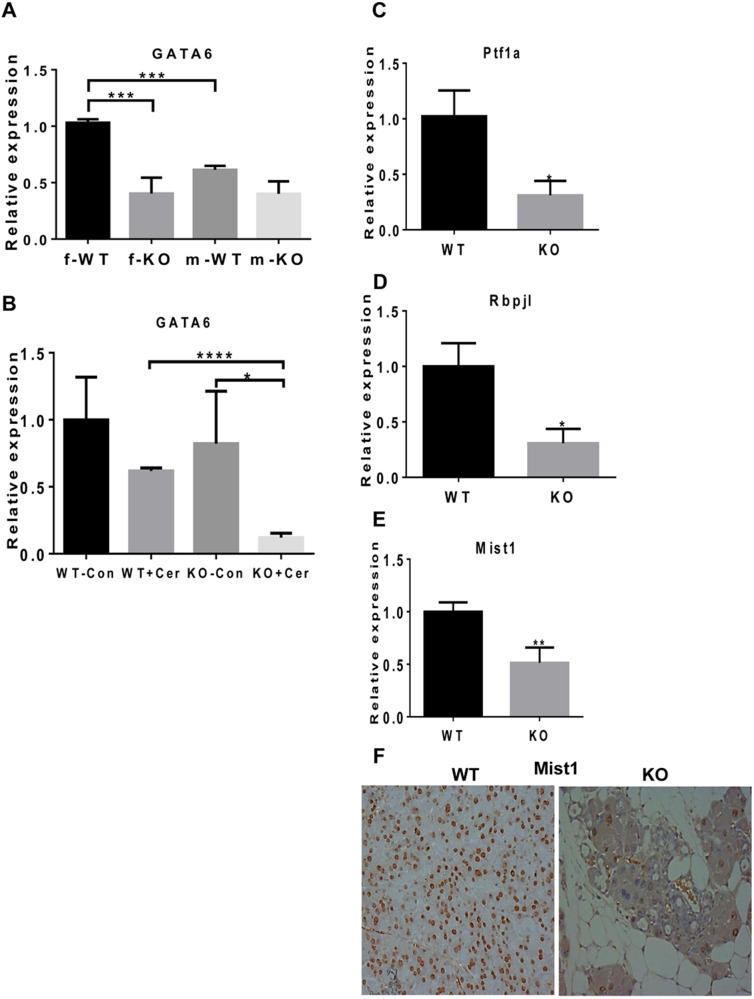
Knockdown of Hpa2 is associated with decreased expression of master regulators of acinar cell differentiation. **A, B** GATA6. Total RNA was extracted from female (f) and male (m) *wt* and Hpa2-KO mice and from *wt* and Hpa2-KO mice administrated with PBS (Con) or cerulein (+CER;). qPCR was then performed applying primers specific to GATA6. GATA6 expression is presented relative to *wt* pancreas, set arbitrarily to a value of 1, and calculated after normalization to actin. **C–E**. qPCR analyses. Total RNA was extracted from *wt* and Hpa2-KO pancreas tissue and was subjected to qPCR analyses applying primers specific for PTF1a, RBPJ1, and Mist1. **F**. Immunostaining. 5-micron sections of *wt* and Hpa2-KO pancreas tissue were subjected to immunostaining applying anti-Mist1 antibody. Shown are representative images at (x50) magnification.

Given that heparanase elevation seems not to be held responsible for the abnormal pancreas of Hpa2-KO mice we sought for other mechanism(s). We suspected that GATA6 or PTF1α may underline the abnormal pancreas morphology of Hpa2-KO mice because the knockout of these genes resulted in phenotypes that closely mimic the Hpa2-KO female phenotype [24, 26, 31]. Notably, qPCR analyses revealed that the expression of GATA6 is 3-fold lower in female Hpa2-KO pancreas vs *wt* control mice (f; Fig. 6A; *p* = 0.0004). We found lower levels of GATA6 expression in the pancreas of male mice, yet comparable GATA6 expression was quantified in *wt* and Hpa2-KO male pancreas (m; Fig. 6A), in agreement with the preserved morphology of male Hpa2-KO pancreas (Fig. 3). However, GATA6 levels were significantly reduced in male Hpa2-KO pancreas following treatment with cerulein (Fig. 6B; *p* = 0.05) in line with the dramatic induction of ADM and accumulation of fat cells following cerulein treatment (Fig. 4). In addition, we found 3-fold lower expression levels of PTF1α in Hpa2-KO female pancreas vs *wt* pancreas (Fig. 6C; *p* = 0.02) and a comparable decrease in the expression levels of Rbpj1 that is tightly regulated by PTF1α (Fig. 6D; *p* = 0.01). Moreover, we found a two-fold decrease in the expression levels of Mist1 (Fig. 6E; *p* = 0.007), which is critically important for full maturation and polarity of acinar cells [32], and this was confirmed by immunostaining (Fig. 6F). Altogether, these results suggest that the abnormal morphology of Hpa2-KO female pancreas involves decreased expression of GATA6, Mist1 and PTF1α, transcription factors that play a critical role in acinar cell differentiation [26, 31].

### Hpa2-KO pancreas is more susceptible to the initiation of pancreatic neoplasia

**Fig. 7:**
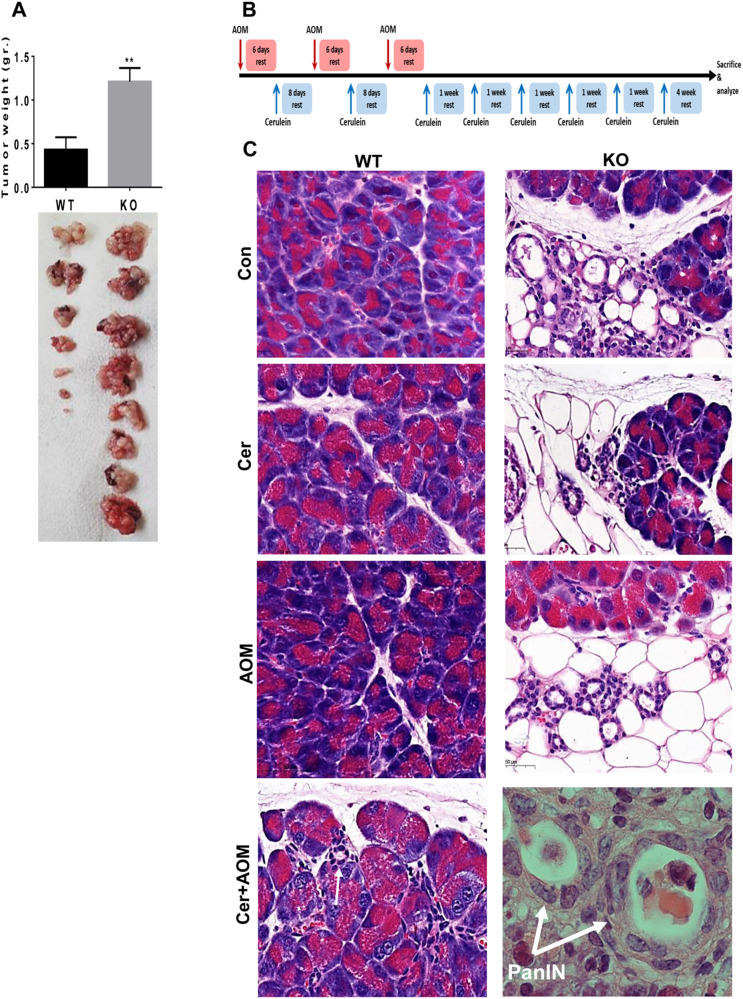
Hpa2-KO pancreas is prone to tumorigenesis. **A** Tumor growth. Panc02 mouse pancreatic carcinoma cells (0.5 × 10^6^) were implanted orthotopically into the pancreas of *wt* (*n* = 8) and Hpa2-KO (*n* = 7) mice. At termination after 4 weeks, mice were sacrificed, tumors were collected, weighed (A, upper panel), and photographed (lower panels). Note that tumors developed in Hpa2-KO mice are 3-fold bigger. **B, C** AOM and cerulein results in pancreatic neoplasia in Hpa2-KO mice. **B**. Schematic diagram of the treatment regimen. **C**. Histology. *wt* and Hpa2-KO mice were treated with PBS (Con), AOM, cerulein (Cer), or both (7 mice per group) as indicated in (**B**). At termination, pancreata were collected, fixed in formalin and embedded in paraffin, and 5-micron sections were subjected to H&E staining. Shown are representative images at ×100 magnification; lower right panel ×250. Note that only Hpa2-KO mice develop PanIN in response to combined treatment with AOM and cerulein (white arrows in the lower right panel). Normal small duct is shown in *wt* mice for comparison (arrow in left lower panel).

In order to reveal the role of Hpa2 in pancreatic tumorigenesis we next implanted mouse Panc-02 pancreatic carcinoma cells orthotopically into the pancreas of control (*wt*) and Hpa2-KO female mice and tumor growth was inspected. Notably, 3-fold bigger tumors were developed in Hpa2-KO pancreas than in control (*wt*) pancreas (Fig. 7A; 0.4 ± 0.1 gr vs 1.2 ± 0.15 gr for *wt* vs Hpa2-KO tumors, respectively; *p* = 0.004), indicating that host-derived Hpa2 is critically important and functions to restrain tumor growth. Increased tumor growth was observed also upon orthotopic implantation of Panc-02 cells into the pancreas of Hpa2-KO male mice. Thus, while only 3/7 *wt* mice developed tumor lesions, all Hpa2-KO (7/7) mice developed tumor lesions that were 2-fold bigger than tumors developed in *wt* mice (not shown).

Since fatty pancreas and ADM are considered to be pro-tumorigenic [27, 33–36], we next exposed *wt* and Hpa2-KO female mice to a carcinogen (AOM) and cerulein, each alone and in combination (Fig. 7B). This treatment regimen was reported to elicit pancreatic neoplasia [37] and is based upon the notion that combining the effect of carcinogens with chronic inflammation elicits tumor initiation and growth [38]. Given the severe morphological alterations of Hpa2-KO pancreas we, nonetheless, reduced the frequency of cerulein treatment from three consecutive days [37] to only once per week (Fig. 7B). AOM and cerulein, alone and in combination, did not elicit noticeable morphological changes in *wt* pancreas (Fig. 7C, left panels). In addition, the abnormal morphology of Hpa2-KO pancreas was not significantly altered by AOM or cerulein, each alone, and the level of fat cells and ADM appeared comparable (Fig. 7C, upper three right panels). However, a combined treatment of Hpa2-KO mice with cerulein and AOM resulted in atypical foci (Suppl. Fig. 6A, upper panel) that were characterized as pancreatic intraepithelial neoplasia (PanIN; Fig. 7C, lower right; Suppl. Fig. 6A), considered to be a histologically well-defined precursor of invasive ductal adenocarcinoma of the pancreas. PanIN was observed in 3/7 Hpa2-KO mice, and at least 2–6 PanIN foci (Suppl. Fig. 6A, upper panel) were detected in each mouse. These results strongly support the notion that Hpa2 functions as a tumor suppressor; in its absence, tissues become more prone to the development of pre-malignant and malignant lesions.

## Discussion

Previously, mutations in HPSE2, considered to result in Hpa2-null phenotype, were identified in urofacial syndrome (UFS), a rare genetic disorder characterized by renal tract syndrome and facial dysmorphology [39]. Interestingly, and unlike the HPSE2 gene trap approach applied for the establishment of HPSE2 mutant mice [23, 40], gross inspection of our conditional Hpa2-KO mice by the voided stain on paper (VSOP) method did not find faults in urination patterns (Suppl. Fig. 6B). This may suggest that urination problems associated with UFS involve deficiency of Hpa2 during embryonic development. Instead, Hpa2 deficiency resulted in profound morphological alterations of the pancreas that becomes more susceptible to the development of pancreatic neoplasia.

Pancreatic ductal adenocarcinoma (PDAC) is currently the fourth leading cause of cancer-related death [41, 42]. The 5-year survival rate at the time of diagnosis is 10% in the USA, because less than 20% of patients are resectable, while others are diagnosed with metastatic disease [42]. Unfortunately, despite intensive efforts to develop new therapeutic modalities, the prognosis of patients with advanced PDAC has only improved by a few months [43]. Therefore, earlier diagnosis and better therapeutic intervention are urgently needed to overcome this aggressive malignancy. Most pancreatic cancers are characterized as ductal adenocarcinoma and thus represent malignancy of the exocrine pancreas, whereas a minority represent neuroendocrine tumors [41, 42]. PDAC arises from precursor lesions, termed pancreatic intraepithelial neoplasia (PanIN), that progress in a stepwise manner through accumulation of mutations that lead to overt adenocarcinoma [42]. Among these are point mutations in the KRas oncogene found in approximately 90% of pancreatic ductal adenocarcinomas [42]. Pre-clinically, it is almost impossible to elicit PDAC in mouse models without bringing active Ras to the pancreas [44]. Here, we show that deficiency of Hpa2 results in fatty pancreas and acinar-to-ductal metaplasia (ADM). This pro-tumorigenic environment not only supports the growth of implanted cancer cells but also leads to the development of pancreatic neoplasia once mice are exposed to conditions that elicit mutations (carcinogen) and prolonged inflammation (cerulein) (Fig. 7). Of note, we found that lack of Hpa2 not only enhances the growth of implanted tumor cells in the pancreas (Fig. 7) but also resulted in far more metastatic lesions in the lungs upon i.v inoculation of mouse B16 melanoma and TC1 lung carcinoma cells (Suppl. Fig. 7). Thus, Hpa2 residing in the host is also critically important to prevent the establishment of metastatic niches in the lung. While the cellular and biochemical nature of these metastatic niches is yet to be resolved, accumulating clinical and pre-clinical evidence supports the pro-tumorigenic properties of fatty pancreas and ADM.

Fatty pancreas was first observed already in the 1930s by imaging studies performed for other indications; it was thought to be an incidental finding and its clinical implications were not thoroughly investigated for several decades [45, 46]. In recent years, however, there is accumulating evidence supporting the association of fatty pancreas with the development of pancreatic cancer as well as other pathologies of the human pancreas [35, 45, 46]. The molecular and cellular mechanisms underlying the pro-tumorigenic properties of adipocytes within the pancreas are thought to involve the tumor microenvironment. Adipocytes secrete many adipokines, cytokines, and chemokines (i.e., IL-1b, TNF-α, IL-6, TGF-β) that affect inflammation and fibrosis which promote cancer development. In addition, imbalances in the endoplasmic reticulum (ER) and angiogenesis are thought to be affected by adipocyte-rich microenvironment [35], features reported to implicate Hpa2 [15, 18, 20].

Intra-pancreatic fat deposition occurs through two mechanisms - fatty replacement (i.e., replacement of acinar cells by adipocytes), or fatty infiltration, the latter mostly associated with obesity and/or metabolic syndrome [35]. Given that Hpa2-KO mice are not obese and seemingly do not exhibit metabolic syndrome(s), the fat accumulating in their pancreas is most likely due to the replacement of acinar cells by fat cells through acinar-to adipocyte-transdifferentiation (AAT) [24, 26], a form of epithelial-to-mesenchymal transition (EMT). Support for the occurrence of this mechanism in the Hpa2 null pancreas is the gradual induction of Pparɣ, which is strongly implicated in adipogenesis [25], shortly after the administration of tamoxifen and the resulting Hpa2 knockout (Fig. 2). The massive EMT that Hpa2-KO acinar cells undergo while transforming into adipocytes strongly implies that Hpa2 functions to restrain EMT. Given the significant role of EMT in cancer progression and metastasis [47], this finding further supports the notion that Hpa2 functions as a tumor suppressor.

Acinar-to-ductal metaplasia (ADM) is thought to facilitate pancreas regeneration after injury, inflammation, or stress and signifies the high degree of plasticity that characterize acinar cells [48]. Normally, ADM is reversible and contributes to the regeneration of acinar structures and repopulation of the pancreas after insults such as injury and stress. However, ADM may become irreversible when cells acquire oncogenic KRas mutations, persistent growth factor signaling, or severe inflammation, which prevent redifferentiation [48]. Under such conditions, ADM may further progress into precancerous pancreatic intraepithelial neoplasia (PanIN) [48, 49]. Although PDACs most often resemble ductal rather than acinar cells, evidence from mouse models pointed to an acinar cell origin in which acinar cells that underwent ductal metaplasia are the ones that give rise to pancreatic tumors [49]. Like AAT, acinar-to-ductal metaplasia (ADM) is thought to reflect a form of EMT in mouse models and human subjects [27]. The spontaneous and massive appearance of ADM in female Hpa2-KO mice (Figs. 1, 2) again implies that Hpa2 functions to maintain the epithelial identity of acinar cells and to prevent EMT. PanIN developed in Hpa2-KO pancreas following AOM/cerulein treatment clearly signifies the pro-tumorigenic nature of Hpa2-deficient pancreas tissue (Fig. 7).

We considered heparanase to be responsible for the abnormal morphology of the Hpa2-KO pancreas. This suspicion emerged from the marked increase in heparanase enzymatic activity in Hpa2-KO pancreas, likely a result of tilting the heparanase: Hpa2 balance in favor of heparanase. This result supports the notion that Hpa2 is a natural inhibitor of heparanase and that the balance between the two is critically important for tissue homeostasis [28–30, 50]. Unexpectedly, however, treating Hpa2-KO mice with the heparanase inhibitors Roneparstat and Pixatimod did not reverse the abnormal morphology of the Hpa2-KO pancreas toward more normal morphology but instead worsened it, resulting in more fat cells and a more ADM (Fig. 5 & Suppl. Fig. 5). The reason for this unexpected result is not entirely clear. However, one interpretation suggests that in the pancreas, heparanase and Hpa2 cooperate and compensate for one another. According to this notion, normal pancreas morphology is maintained in the absence of heparanase (heparanase-KO mice) because Hpa2 compensates for heparanase deficiency (not shown); heparanase can compensate, to some extent, for Hpa2 deficiency, whereas deficiency of both Hpa2 (Hpa2-KO) and heparanase activity (heparanase inhibitors), is most devastating to the exocrine pancreas. It should be kept in mind that the heparanase inhibitors employed in our study are HS mimetics that not only inhibit heparanase enzymatic activity but also its interaction with cellular HS [51, 52]. It is thus possible that interaction with HS, common to both heparanase and Hpa2, is the biological cue critical for acinar cells' identity. HS was shown to play a critical role in the endocrine aspect of the pancreas (beta cells) [53]. The current study may suggest that HS levels, or the interaction of heparanase/Hpa2 with HS play an important role also in acinar cells. In addition to the capacity of Hpa2 to inhibit the enzymatic activity of heparanase, the two proteins were found to be physically associated with one another [11]. This physical association possibly affects the cellular localization of heparanase. This possibility emerges from studies showing that heparanase can reach the nucleus and promote cell differentiation [54], trafficking that may be affected by the interaction of heparanase with other molecules, including Hpa2. Such a mechanism awaits detailed confirmation.

For many years, an intensive effort was dedicated to revealing the role of heparanase in human pathologies, mainly in cancer, leading to the appreciation that heparanase is a target for the development of anti-cancer therapeutics [55]. However, the role of heparanase in normal, non-transformed, cells is still largely unknown. The present study suggests, for the first time, that heparanase plays a critical role in the exocrine aspect of the pancreas, cooperating with Hpa2 to maintain the differentiation state and function of acinar cells.

Searching for other mechanisms that may underlay the severe morphological abnormalities of the Hpa2-KO pancreas, we found that the expression of PTF1, GATA6, and MIST1, master transcription factors implicated in acinar cell differentiation, is significantly reduced in Hpa2-deficient pancreas (Fig. 6). Pancreas transcription factor 1 (PTF1) plays a fundamental role in maintaining the differentiation state of acinar cells [31, 56, 57]. Furthermore, knockdown of PTF1 is sufficient to induce ADM, potentiate inflammation and accelerate development of invasive PDAC by sensitizing cells to KRas-mediated transformation [48]. Notably, knockdown of PTF1 results in apoptosis of acinar cells by activation of ER stress pathway [58], thus further linking Hpa2 with ER stress responses [18]. Another important player in acinar cell differentiation is GATA6. This transcription factor functions to maintain acinar cell differentiation by suppressing pro-inflammatory and EGFR signaling pathways [59]. GATA6-KO mice exhibit extensive ADM and accumulation of adipocytes and, in the context of an active KRas (KRas*;Gata6^-/-^ mice), accelerated tumor development [59]. Therefore, like PTF1, GATA6 functions as a tumor suppressor in the pancreas [56, 59]. The capacity of Hpa2 to regulate the expression of key regulators of acinar cell differentiation such as PTF1, GATA6, and MIST1 [32] (Fig. 6) grant further support for the critical role of Hpa2 in the exocrine pancreas and its tumor suppressor characteristics.

Collectively, our results strongly implicate Hpa2 in acinar cell differentiation and homeostasis; deficiency of Hpa2 results in pre-neoplastic pancreas which, in response to further insults, develops into pancreatic neoplasia. We hope that the protective effects of Hpa2 against cancer and inflammation will be translated to the development of Hpa2-based therapeutic strategies.

## Materials and methods

### Conditional Hpa2-KO mice

HPSE2^fl^ mice (C57BL/6) were generated by Cyagen. Briefly, for the generation of the HPSE2 conditional knockout allele (HPSE2^fl^), the Neo cassette was flanked by SDA (self-deletion anchor) sites and the cKO region was flanked by loxP sites, directed to the introns flanking exon 5. Homologous recombination was verified via PCR and Southern blot techniques. B6.Cg-Tg (CAG-cre/Esr1*) 5Amc/J mice were purchased from JAX mice (JAX Stock No:004682). These CAGGCre-ERTM transgenic mice have a tamoxifen-inducible Cre-mediated recombination system driven by the chicken beta-actin promoter/ enhancer coupled with the cytomegalovirus (CMV) immediate-early enhancer. When bred with mice containing loxP-flanked sequences, tamoxifen-inducible Cre-mediated recombination results in deletion of the floxed sequences in widespread cells/tissues of the offspring. Cre activation was performed via administration (i.p) of tamoxifen (0.1 ml; Sigma T5648, 20 mg/ml dissolved in corn oil) every day for 5 days, resulting in the removal of exon 5 and the disruption of HPSE2 open reading frame. Tamoxifen was administrated to 5–6 weeks-old Cre^+^ mice, and to age-matched control C57BL/6 mice. HPSE2 gene editing by Cre activation was validated by PCR. For experiments, we utilized 3–4 month-old mice (typically 6–8 weeks following the administration of tamoxifen), or mice at the time indicated following the administration of tamoxifen.

### Histopathological analyses

Mouse pancreata, collected from at least 7 mice, were fixed with 4% paraformaldehyde and embedded in paraffin using standard protocol. The paraffin-embedded sections (5 μm) were stained with hematoxylin and eosin (H&E), Masson’s trichrome, and oil red as described below. Specimens were evaluated and scored by an expert pathologist, in a blinded manner. A total of at least five high-power fields in each pancreatic section, prepared from the pancreata of 5–7 mice, were evaluated for acinar cell hypertrophy, edema, acinar-to-ductal metaplasia (ADM), acinar-to-adipocytes transdifferentiation (AAT), and pancreatic intraepithelial neoplasia (PanIN).

### Oil red and alcian blue staining

Pancreas tissues were fixed in formalin for 24 h and were then placed in 30% sucrose until the pancreas sinks to the bottom of the tube (24 h and up to 4 days). Samples were then embedded in OCT and snap-frozen on dry ice. Frozen sections were subjected to Oil Red O (dissolved in 85% propylene glycol) staining by established protocol. Oil red staining intensity was quantified by Image Pro software.

Histochemical localization of heparan sulfate in mouse pancreas by alcian blue staining (pH 5.8) was carried out according to a detailed protocol [60] and the staining was similarly quantified by the Image Pro software.

### Antibodies and reagents

Anti-amylase (sc-166349), anti-Ppar ɣ (sc-7273) and anti-cytokeratin 19 (sc-376126) antibodies were purchased from Santa Cruz Biotechnology; anti-Sox9 (#82630), anti-Pan-Keratin (C11; #4545), and anti-Mist1 (#14896) antibodies were purchased from Cell Signaling; anti-Ki67 (ab833) antibody was purchased from Abcam; anti CD45R (a B cell marker) was purchased from BioLegend (cat:103206) and anti F4/80 (a marker of mouse macrophages) antibody was from Bio-Rad. Anti-actin (clone AC-74) and anti-smooth muscle actin (SMA; clone 1A4) antibodies, tamoxifen, cerulein, azoxymethane (AOM), alcian blue 8GX, and safranin were purchased from Sigma. The heparanase inhibitors Roneparstat (SST0001) and Pixatimod (PG545) were kindly provided by Leadiant Biosciences S.p.A, Rome, Italy, and Zucero Therapeutics Ltd, Darra, Queensland, Australia, respectively [61, 62]. A proteome profiler mouse cytokine antibody array (ARY028) was purchased from R&D systems.

### Experimental pancreatitis associated pancreatic neoplasia

Chronic pancreatitis **(**CP) was induced by repetitive intraperitoneal (i.p) administration of cerulein without or with azoxymethane (AOM), each alone and in combination, as described elsewhere [37]. AOM (10 mg/kg) was administrated i.p in 0.1 ml saline; Cerulein (50 μg/kg) was given by repetitive 6 hourly i.p injections, in 0.1 ml saline, as reported [37].

### Cells, cell culture, and immunoblotting

Mouse Panc-02 pancreatic carcinoma and B16 melanoma cells have been described previously [63]. Luciferase-labeled mouse TC1 lung carcinoma cells were kindly provided by Dr. T-C Wu (Johns Hopkins University, Baltimore, MD, USA) [64]. Cells were grown in Dulbecco’s modified Eagle’s medium (Biological Industries, Beit Haemek, Israel) supplemented with 10% fetal bovine serum and antibiotics. Cells were passed in culture no more than 2 months after being thawed from authentic stocks. Preparation of cell/tissue lysates and immunoblotting was performed essentially as described [18]. Cells were found negative for mycoplasma.

### Tumorigenicity and immunohistochemistry

Panc-02 cells were detached with trypsin/EDTA, washed with PBS, and brought to a concentration of 1 × 10^8^ cells/ml. Cell suspension (0.5 × 10^6^/0.01 ml) was inoculated subcapsularly to the pancreas of 12–14 week-old female Hpa2-KO mice, essentially as described [18]. At termination, mice were sacrificed and tumor xenografts were removed and weighed; Small portions were taken for the extraction of RNA and proteins, and the other portion was fixed in formalin for histological examination. B16 and luciferase-labeled TC1 cells (1 x 10^5^) were injected into the tail vein of *wt* and Hpa2-KO mice, and TC1 metastases growth was inspected by IVIS, essentially as described [65]. At termination, lungs were collected, fixed in Bouin’s solution, and the number of metastases was counted under a binocular. All experiments were performed in accordance with the Technion’s Institutional Animal Care and Use Committee (IL-078-05-2021; OPRR-A5027-01).

### Real time-PCR

Real time-PCR analyses were performed using ABI PRISM 7000 Sequence Detection System employing SYBR Green PCR Master Mix (Applied Biosystems, Foster City, CA), essentially as described [18]. The primer sets utilized in this study are summarized in Suppl. Table 1.

### Heparanase activity assay

Preparation of dishes coated with sulfate labeled ECM and determination of heparanase enzymatic activity (i.e., release of sulfate labeled HS degradation fragments) were carried out essentially as described previously [65]. Briefly, extracts were prepared by tissue homogenization in phosphate/citrate buffer (pH 6.0) followed by three cycles of freeze (liquid nitrogen)/thaw (37 °C), and 200 µg protein were incubated (18 h, 37 °C, pH 6.0) with the labeled ECM. Sulfate-labeled degradation fragments released into the incubation medium were analyzed by gel filtration on a Sepharose CL-6B column, as described [65]. Degradation fragments of HS side chains are eluted from Sepharose 6B at 0.5<Kav<0.8 (fractions 10–30).

Heparanase activity, qPCR and immunoblotting analyses were performed utilizing protein/RNA extracts prepared from a pool of pancreas tissues, collected from at least 5 mice.

### Statistics

Data are presented as means ± SEM. Statistical significance was analyzed by 2-tailed Student’s *t*-test. Values of *p* ≤ 0.05 were considered significant and designated as follow: ^*^p ≤ 0.05, ^**^p ≤ 0.01, ^***^p ≤ 0.001, ^****^p ≤ 0.0001. Data sets passed D'Agostino-Pearson normality (GraphPad Prism 5 utility software).

All experiments were performed in accordance with the Technion’s Institutional Animal Care and Use Committee (IL-078-05-21; OPRR-A5026-01).

## Supplementary information

Suppl. Table 1

Suppl. Figure legends

Suppl. Figure 1

Suppl. Figure 2

Suppl. Figure 3

Suppl. Figure 4

Suppl. Figure 5

Suppl. Figure 6

Suppl. Figure 7

Original Data file
