# Supplementary material for: Heparanase 2 (Hpa2)- a new player essential for pancreatic acinar cell differentiation
Source: Cell Death Dis. 2023 Jul 25;14(7):465. doi: 10.1038/s41419-023-05990-y (PMC10368643; doi:10.1038/s41419-023-05990-y)
Supplement: Supplementary file 1 — Suppl. Table 1 [file 41419_2023_5990_MOESM1_ESM.docx]

| **Primer** | **Forward**  **Sequence 5'-3** | **Reverse**  **Sequence 5'-3** |
| --- | --- | --- |
| Mouse | | |
| IL-8 | CACCTCAAGAACATCCAGAGCT | CAAGCAGAACTGAACTACCATCG |
| IL-10 | GCTCTTACTGACTGGCATGAG | CGCAGCTCTAGGAGCATGTG |
| TNFα | TCAGCCTCTTCTCATTCCTG | TGAAGAGAACCTGGGAGTAG |
| TGFβ | CAACAATTCCTGGCGTTACCTTGG | GAAAGCCCTGTATTCCGTCTCCTT |
| Hpa1 | ACCGACGACGTGGTAGACTT | TGCAGGAGATAAGCCTCTAGC |
| Hpa2 | CCTGGAACAGTTCTAGTGCCC | ATGCTCCGATAGTTATTTGGCTC |
| GATA6 | CTCTGCACGCTTTCCCTACT | GACAGCGAGCTGTACTGGTG |
| Ptf1a | AACCAGGCCCAGAAGGTTAT | AAAGAGAGTGCCCTGCAAGA |
| Rbpjl | CAGCAAAGATGTGAGCAGACT | GGTAGACACAGGGTGGAGGA |
| Mist1 | AGGGAGTGATCTGGGCCTTC | CTGGAGTCGTCCCTTAGCCA |
| GAPDH | AACTTTGGCATTGTGGAAGG | ACACATTGGGGGTAGGAACA |
| Actin | ATGCTCCCCGGGCTGTAT | CATAGGAGTCCTTCTGACCCATTC |

**Suppl. Table 1**. Primer sets employed in this study
