# Supplementary material for: Heparanase 2 (Hpa2)- a new player essential for pancreatic acinar cell differentiation
Source: Cell Death Dis. 2023 Jul 25;14(7):465. doi: 10.1038/s41419-023-05990-y (PMC10368643; doi:10.1038/s41419-023-05990-y)
Supplement: Supplementary file 2 — Suppl. Figure legends [file 41419_2023_5990_MOESM2_ESM.docx]

**Suppl. Figure legends**

**Suppl. Figure 1**. Hpa2 expression is prominently decreased in tissues derived from Hpa2-KO mice. **A**. A schematic diagram of HPSE2 introns (yellow)/exons (red) composition. loxP sites were directed to the introns flanking exon 5, resulting in deletion of this exon and disruption of the open reading frame. Decreased Hpa2 mRNA levels in Hpa2-KO mice are expected due to a nonsense-mediated decay mechanism. **B**. qPCR. Total RNA was extracted from the indicated tissue harvested from *wt* and Hpa2-KO mice and subjected to qPCR applying a primer set specific for HPSE2. HPSE2 expression in tissues collected from Hpa2-KO mice is presented relative to *wt* mice, set arbitrarily to a value of 1, and calculated after normalization to actin.

**Suppl. Figure 2.** Kinetic analyses. Pancreata were collected from *wt* (Cre^-^), Cre^+^, and Cre^+^ mice 1, 2, 3, 4, and 6 weeks after the administration of tamoxifen. 5-micron sections of formalin-fixed, paraffin-embedded pancreas tissues were subjected to H&E staining. Shown are representative images at x100 magnification. Corresponding pancreas extracts were subjected to immunoblotting applying anti-cytokeratin 19 (CK19) and anti-actin (lower right panels) antibodies.

**Suppl. Figure 3**. **A**. Plin expression. Total RNA was extracted from *wt* and Hpa2-KO pancreata (n=5) and was subjected to qPCR analysis applying primers specific for Plin1 and Plin4. **B**. Expression of many cytokines is increased in the pancreas of Hpa2-KO male mice. Pancreata were collected from 3-month-old *wt* and Hpa2-KO mice (n=5) and tissue extracts were applied onto a cytokine antibody array according to the manufacturer's (R&D Systems) instructions. Band intensities observed on the membranes array (left panels) were quantified by densitometry and presented as fold-increase in Hpa2-KO vs *wt* pancreas (right panel). **C**. Heparanase activity. Pancreas tissue was collected from *wt* and Hpa2-KO male mice (n=5), homogenized, and applied onto dishes coated with ^35^S-labelled ECM, as described under 'Materials and Methods'. Note increased heparanase activity in Hpa2-KO pancreas.

**Suppl. Figure 4**. Histological analyses. Pancreas was collected from control (*wt*) and Hpa2-KO male mice untreated (Con) or treated with cerulein (+Cer). 5-micron sections were subjected to immunostaining applying anti-cytokeratin 19 (upper panels), anti-Sox9 (second panels) or anti-F4/80 (lower panels; macrophages) antibodies. Shown are representative images at original magnifications of x 100.

**Suppl. Figure 5**. Heparanase inhibitors do not reverse the morphology of Hpa2-KO pancreas. Histological evaluation. *wt* and Hpa2-KO mice (n=6) were administrated with PBS (Con) or Pixatimod (PG545; 400 µg/mouse). One week thereafter, pancreata were collected, fixed in formalin and embedded in paraffin. 5-micron sections were subjected to H&E staining. Shown are representative images at original low (x10; upper panels) and high (x25; middle panels) magnifications. Representative images of oil red staining are shown at the lower panels, and quantification of the oil red staining intensity is shown graphically in the lower right panel.

**Suppl. Figure 6**. **A**. Histology. H&E staining of Hpa2-KO pancreas following treatment with AOM and cerulein. Note a foci of PanIN (upper panel). Shown are representative images at x100 (upper panel) and x250 (middle and lower panels) Magnification**s**. **B**. Voided stain on paper (VSOP) method. *wt* and Hpa2-KO mice (n=6) were placed individually in each cage for 3h and urine drops, indicating urination pattern, were collected. At the end of the assay period, filter paper was retrieved and urine spots left behind were imaged under ultraviolet light. Note that the urination pattern appeared comparable among *wt* (upper panels) and Hpa2-KO (lower panels) mice.

**Suppl. Figure 7**. Hpa2 mediates the establishment of lung metastatic niches. Mouse B16 melanoma cells (1.5x10^6^) (**A**) and Luciferase-labeled mouse TC1 lung carcinoma cells (1x10^6^) (**B**) were inoculated (i.v) into the tail vein of *wt* and Hpa2-KO mice (n=8). 4 weeks thereafter, lungs were collected and lung metastases were visualized (A, left panel) and counted (A, right panel); Lung metastases of TC1 cells were visualized by IVIS (B, upper panel) and by gross inspection (B, lower panel). Quantification of the IVIS measurements is shown graphically in (B) right panel.
