# Supplementary figures and images for: Heparanase 2 (Hpa2)- a new player essential for pancreatic acinar cell differentiation
Source: Cell Death Dis. 2023 Jul 25;14(7):465. doi: 10.1038/s41419-023-05990-y (PMC10368643; doi:10.1038/s41419-023-05990-y)

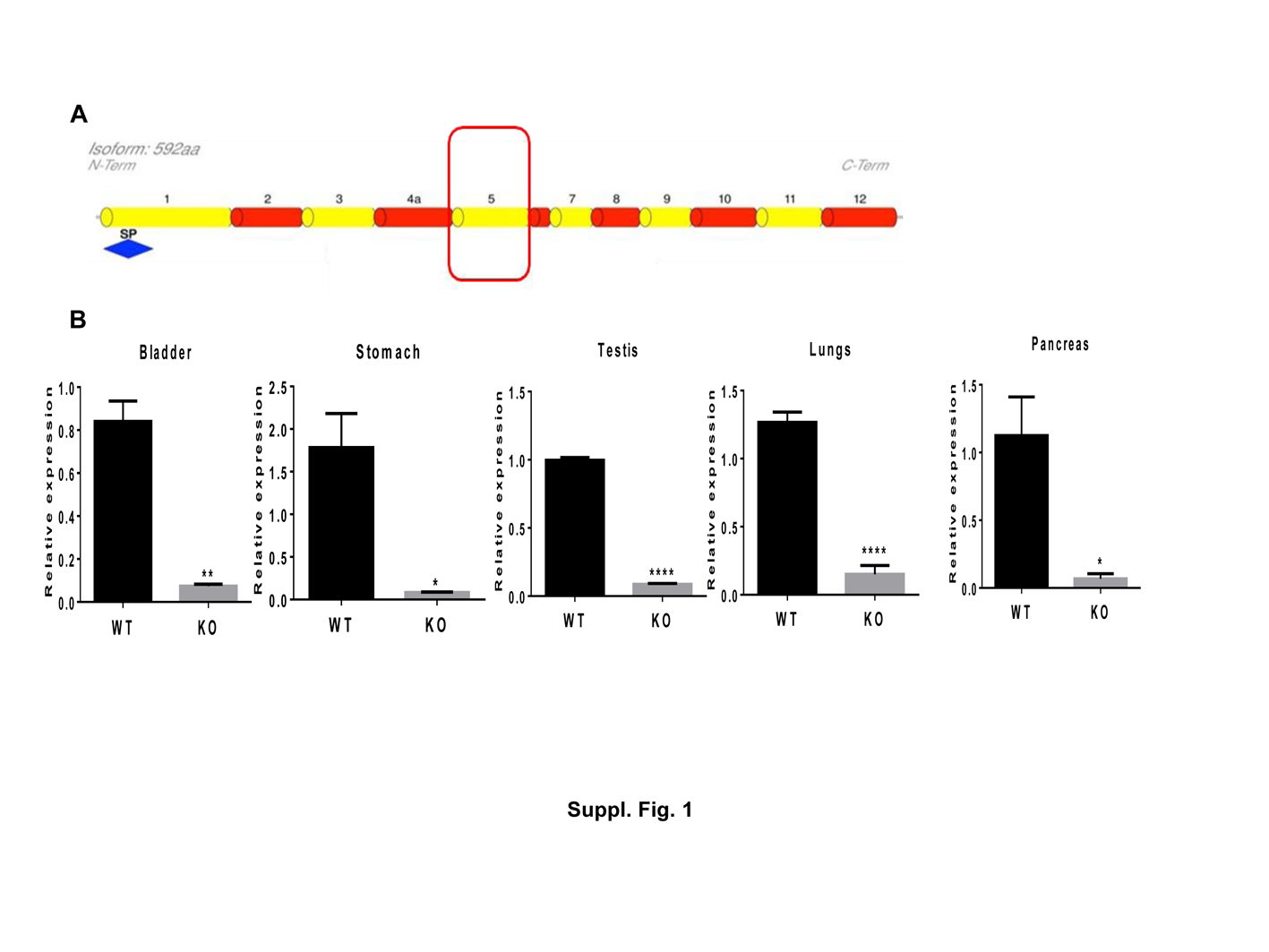

Supplement: Supplementary file 3 — Suppl. Figure 1 [file 41419_2023_5990_MOESM3_ESM.tif]

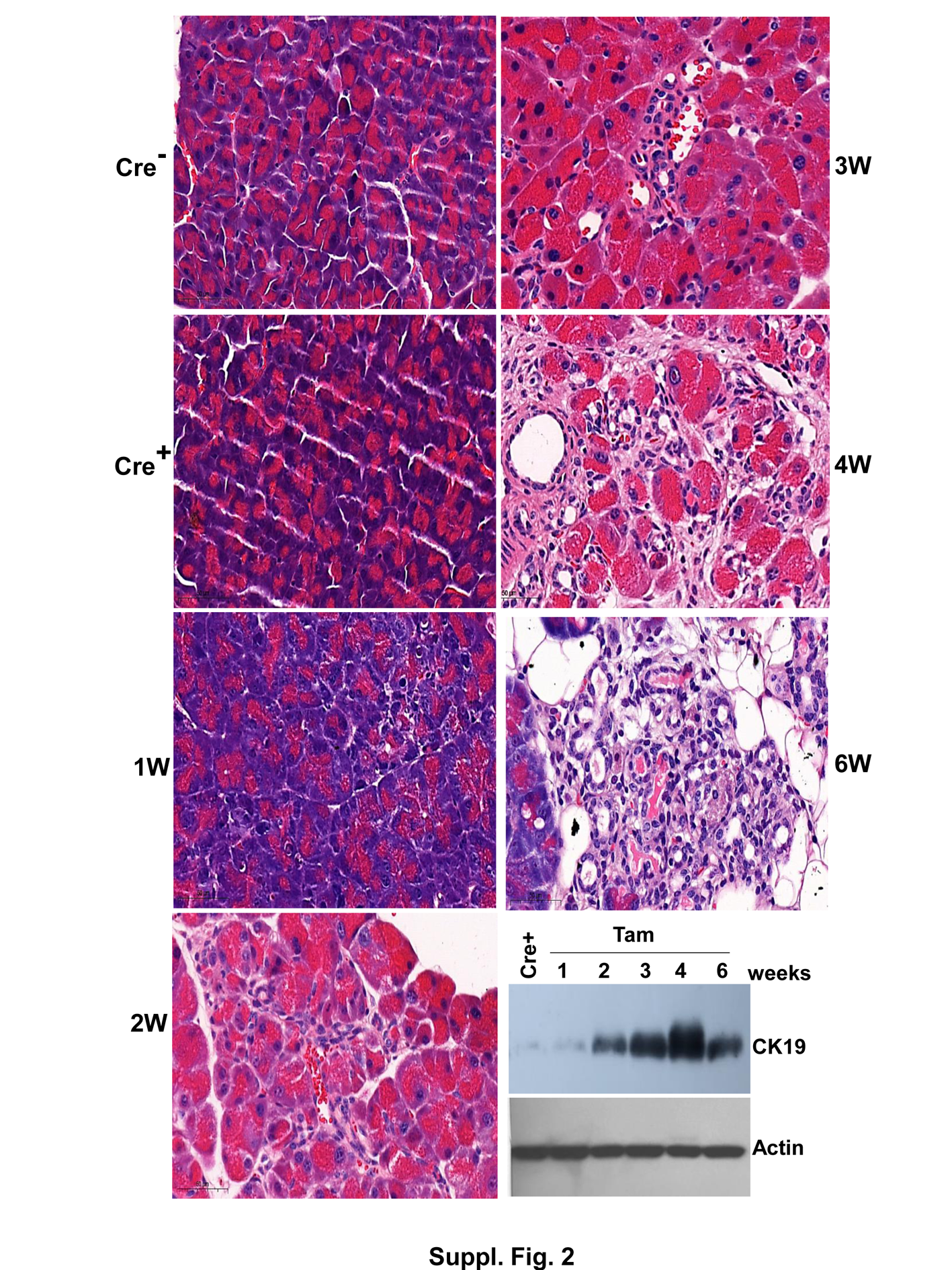

Supplement: Supplementary file 4 — Suppl. Figure 2 [file 41419_2023_5990_MOESM4_ESM.tif]

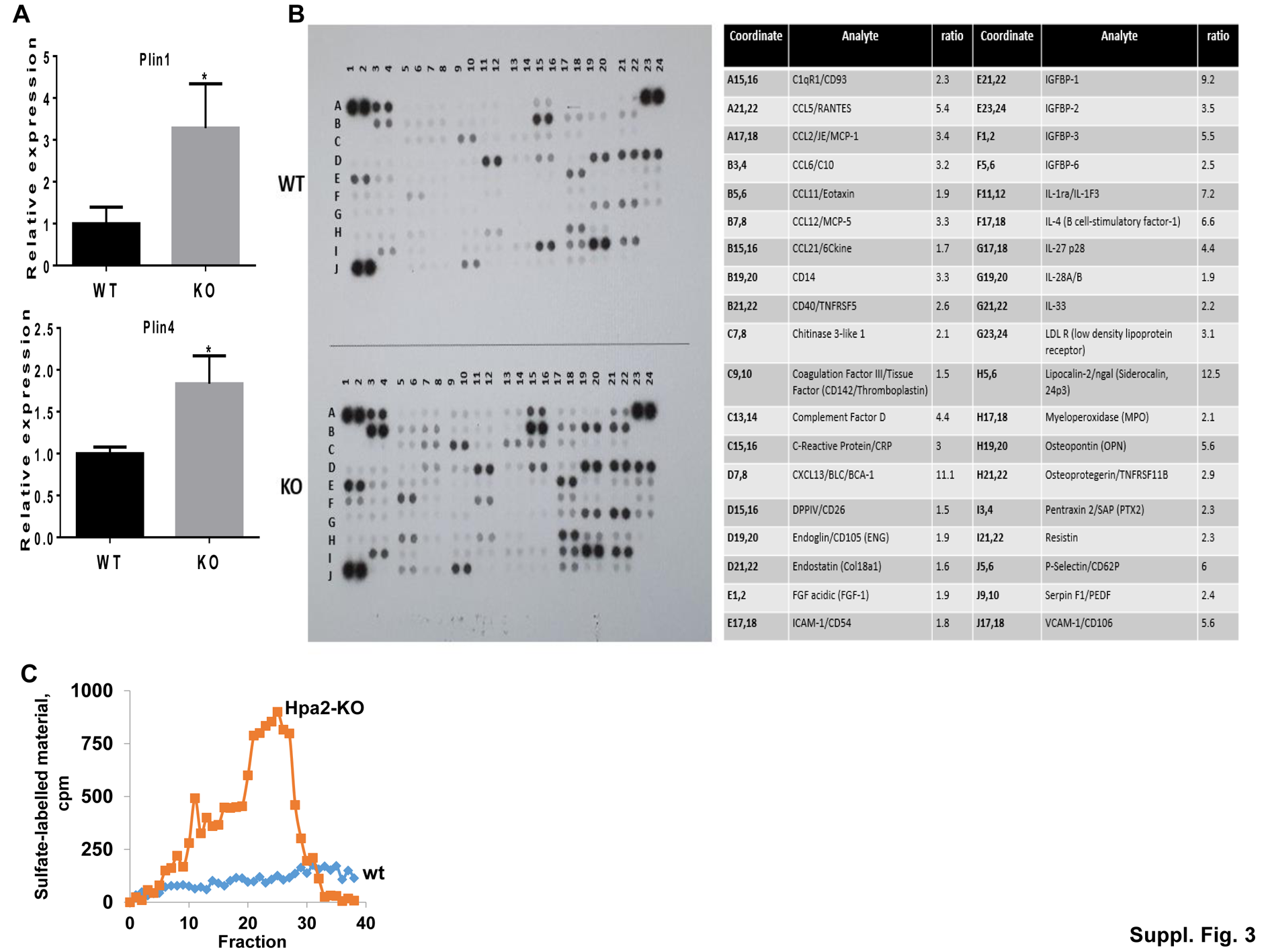

Supplement: Supplementary file 5 — Suppl. Figure 3 [file 41419_2023_5990_MOESM5_ESM.tif]

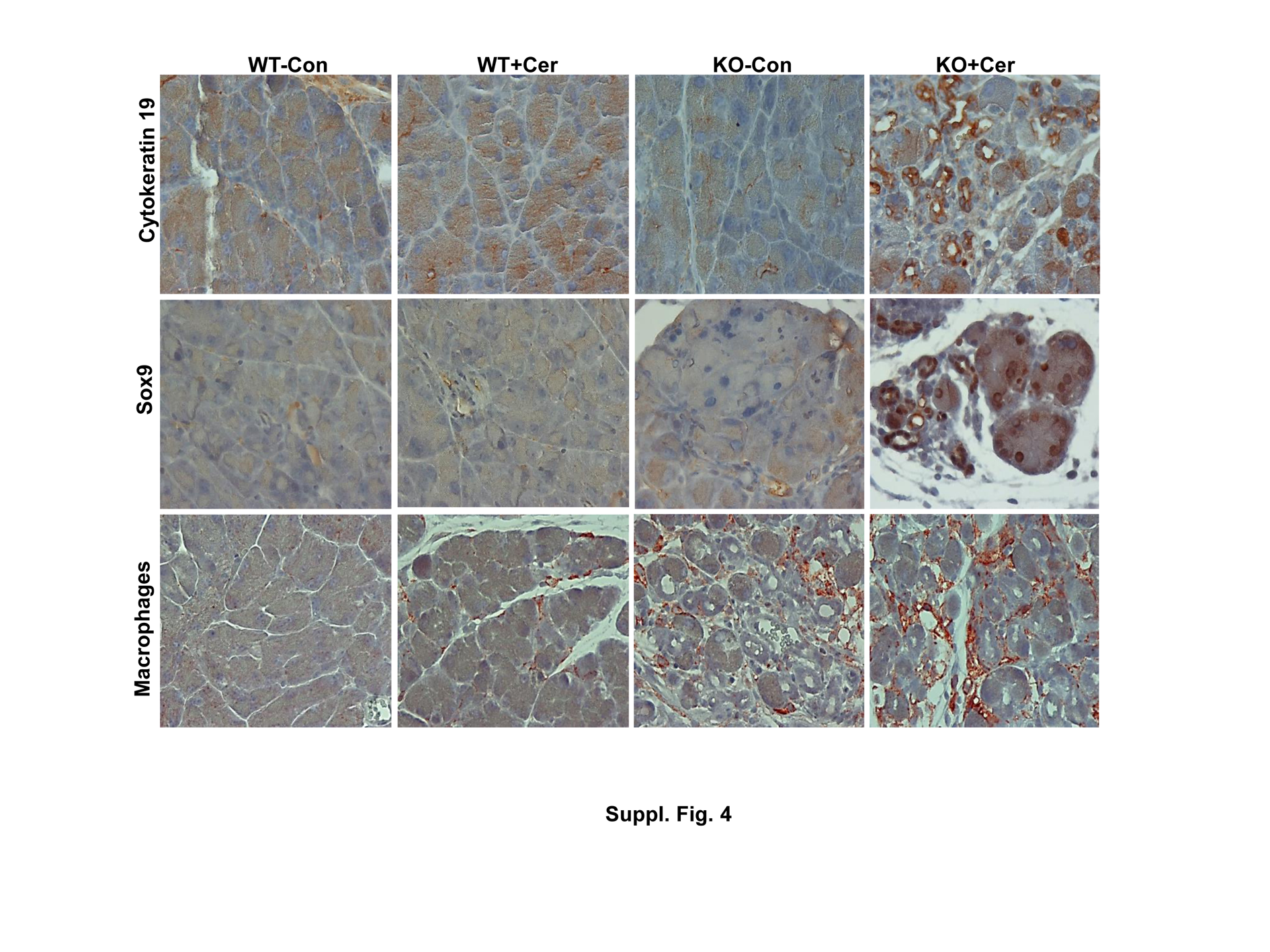

Supplement: Supplementary file 6 — Suppl. Figure 4 [file 41419_2023_5990_MOESM6_ESM.tif]

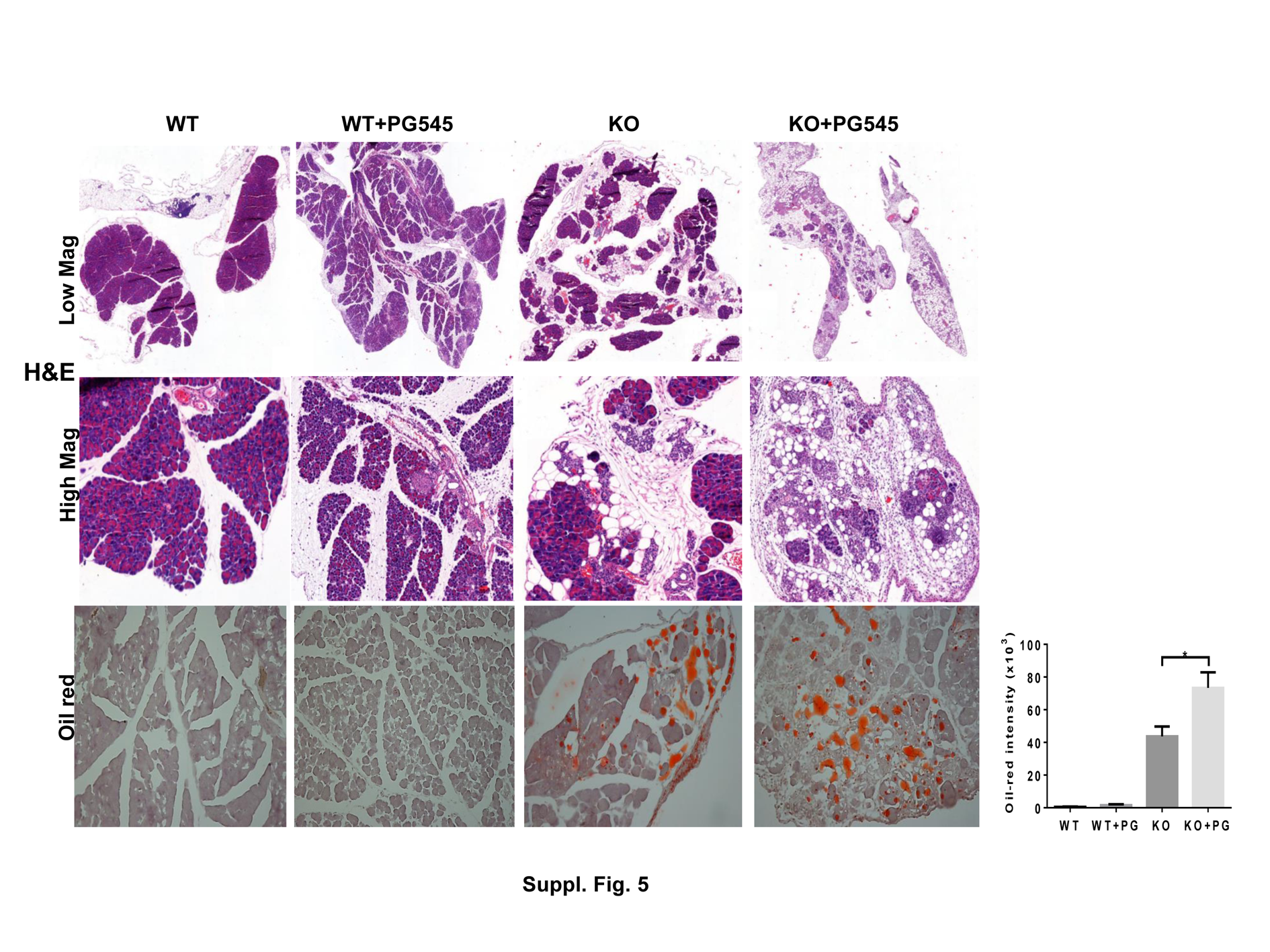

Supplement: Supplementary file 7 — Suppl. Figure 5 [file 41419_2023_5990_MOESM7_ESM.tif]

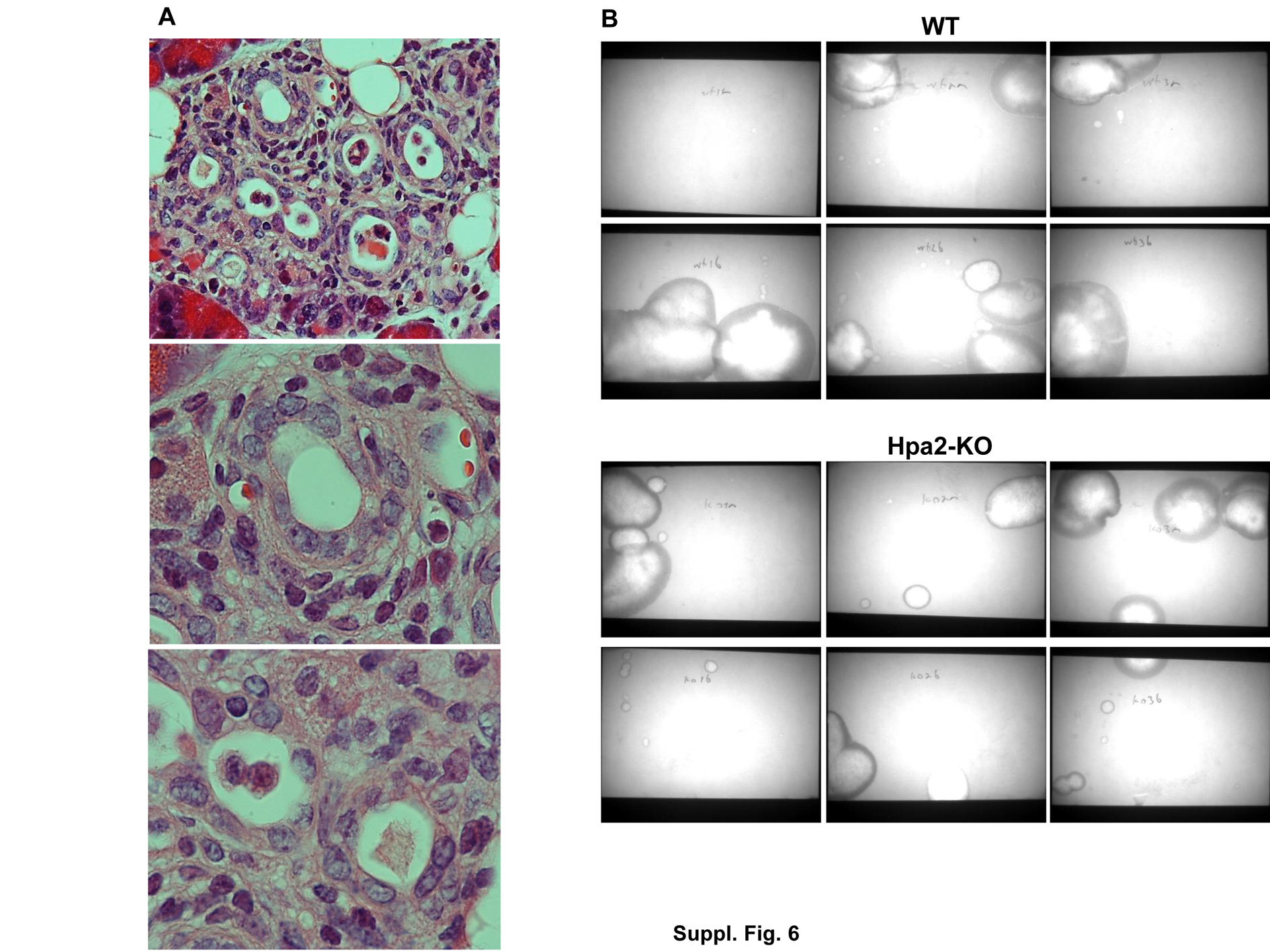

Supplement: Supplementary file 8 — Suppl. Figure 6 [file 41419_2023_5990_MOESM8_ESM.tif]

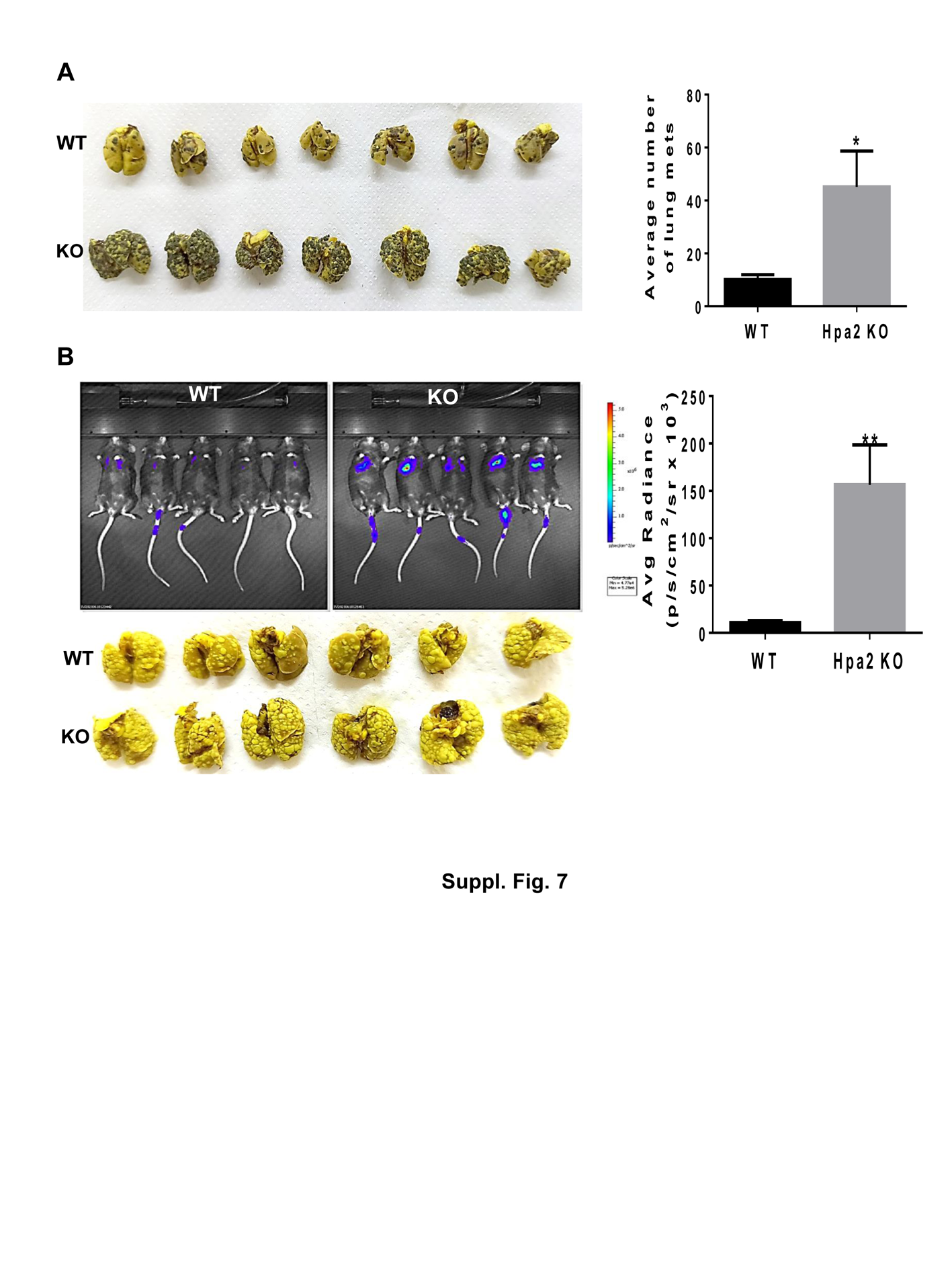

Supplement: Supplementary file 9 — Suppl. Figure 7 [file 41419_2023_5990_MOESM9_ESM.tif]
