# Supplementary material for: Heparanase 2 (Hpa2)- a new player essential for pancreatic acinar cell differentiation
Source: Cell Death Dis. 2023 Jul 25;14(7):465. doi: 10.1038/s41419-023-05990-y (PMC10368643; doi:10.1038/s41419-023-05990-y)
Supplement: Supplementary file 10 — Original Data file [file 41419_2023_5990_MOESM10_ESM.pdf]

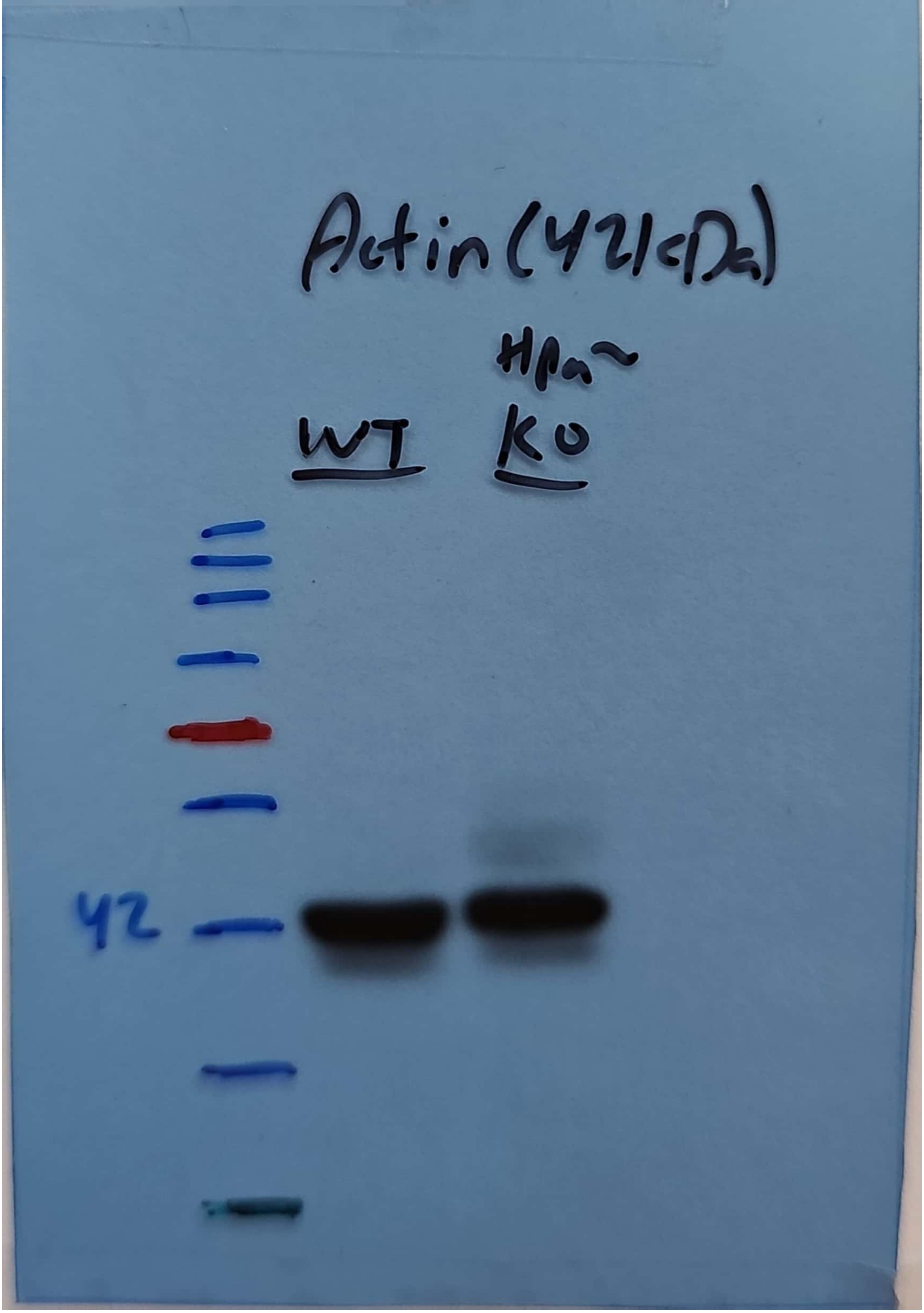

CamScanner

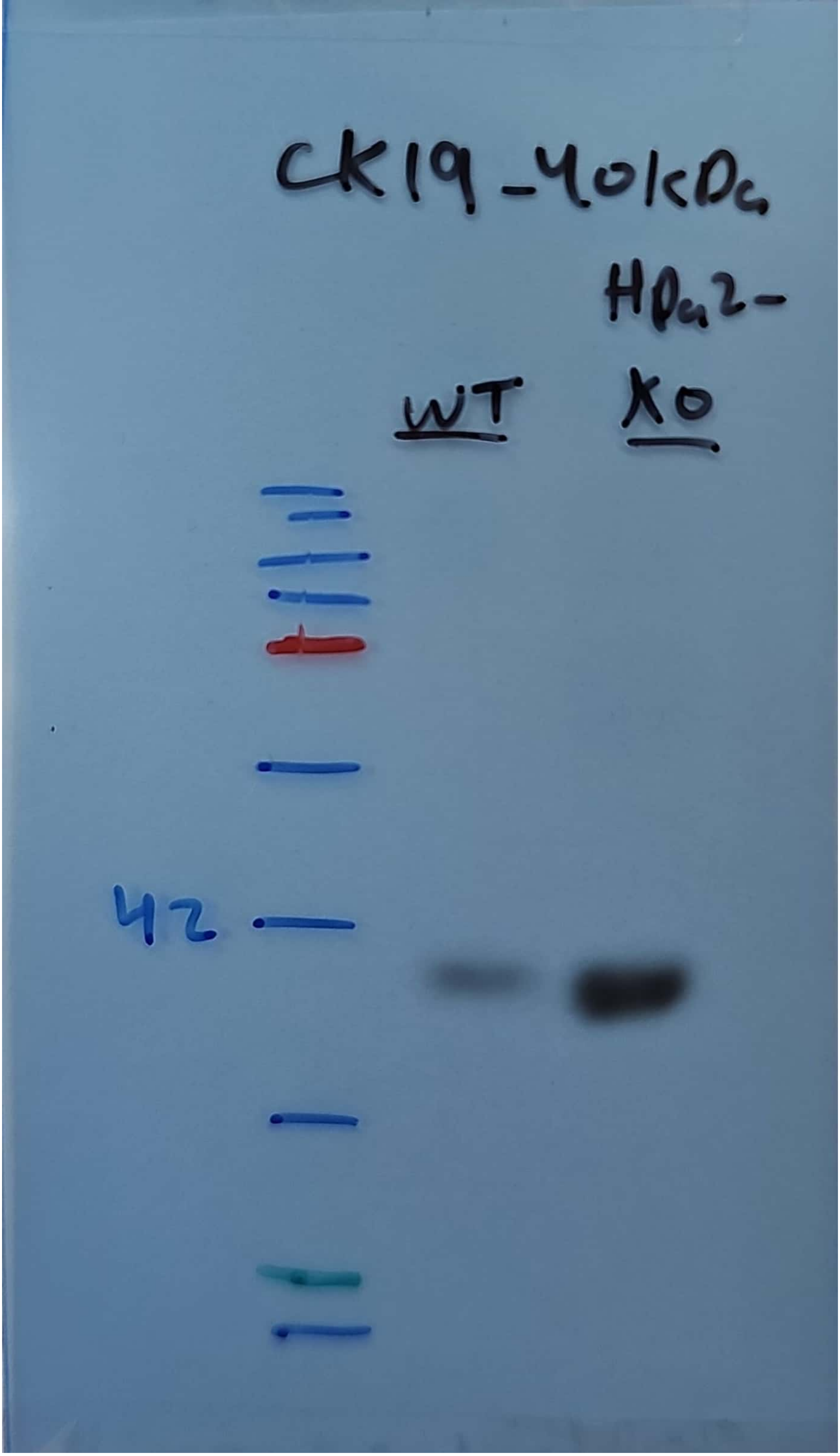

CS CamScanner

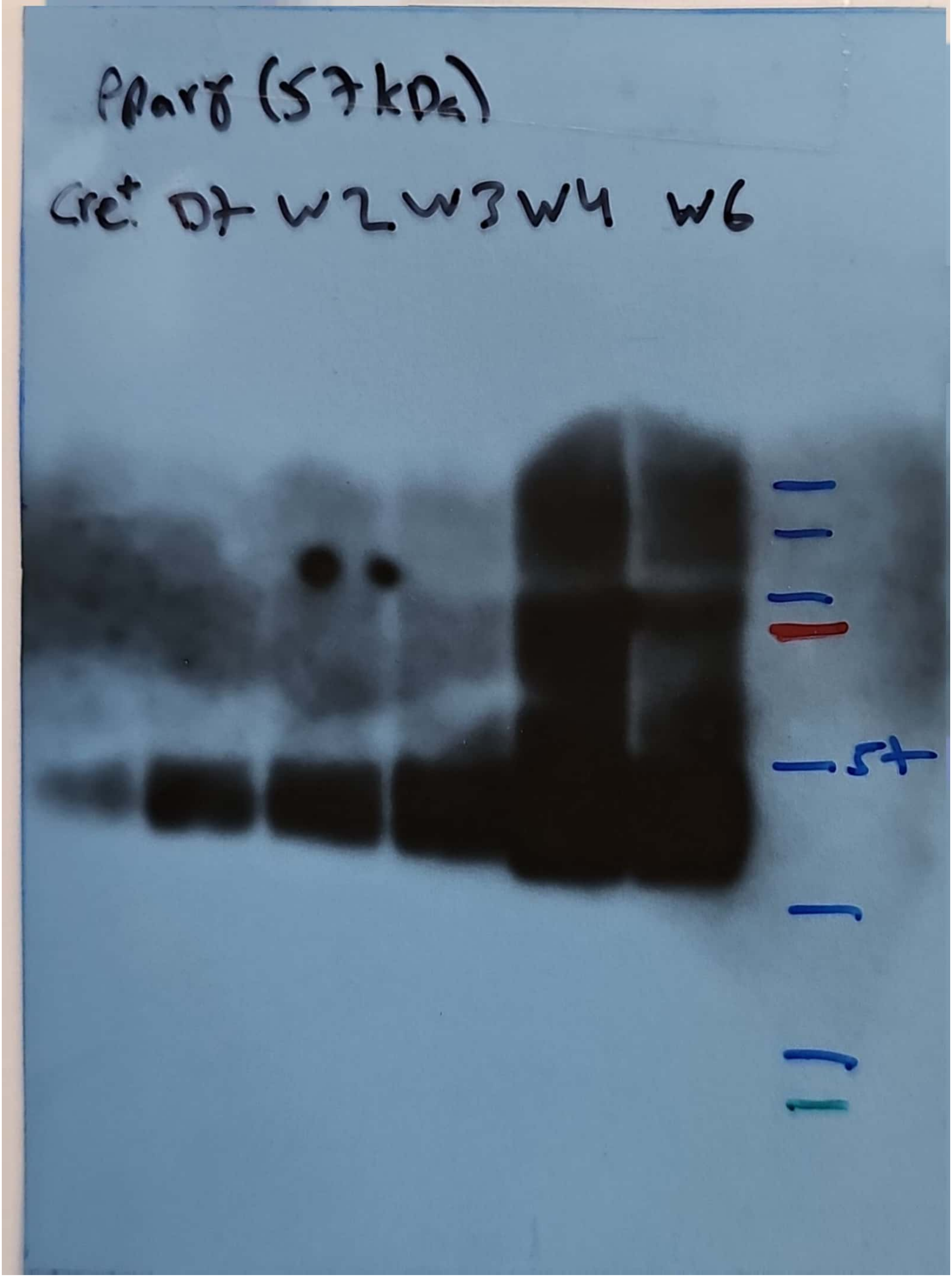

CamScanner

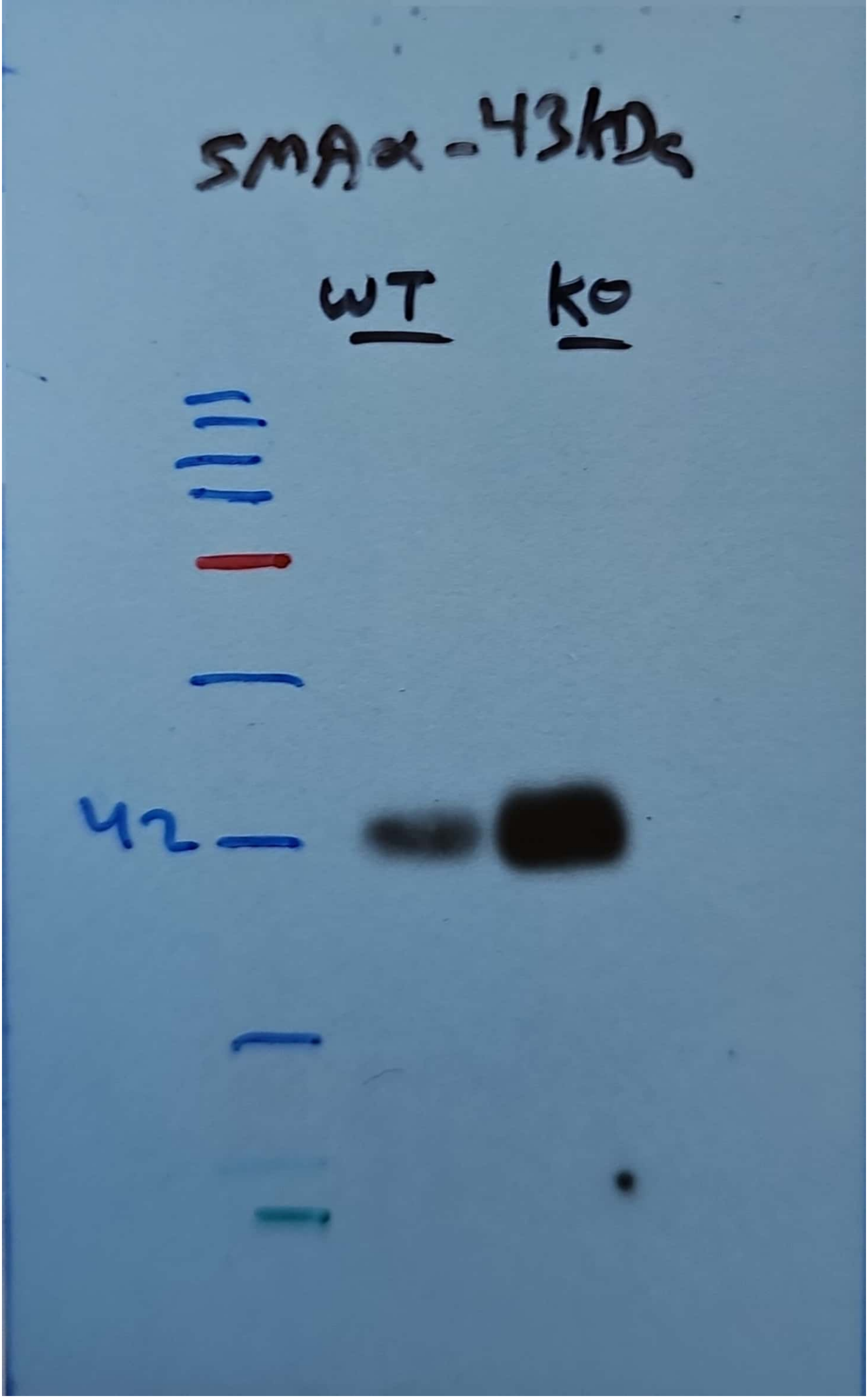

CamScanner

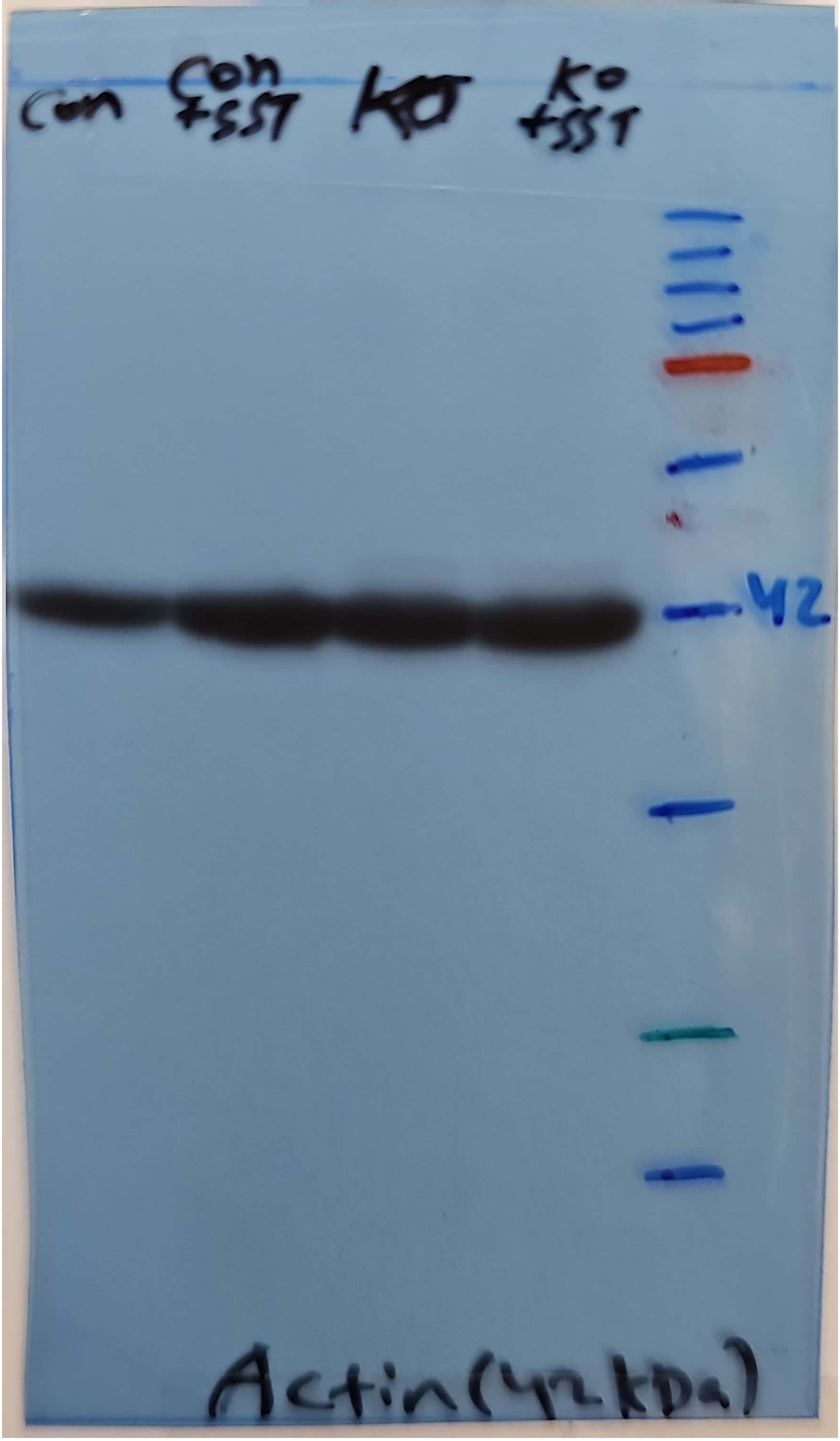

CamScanner

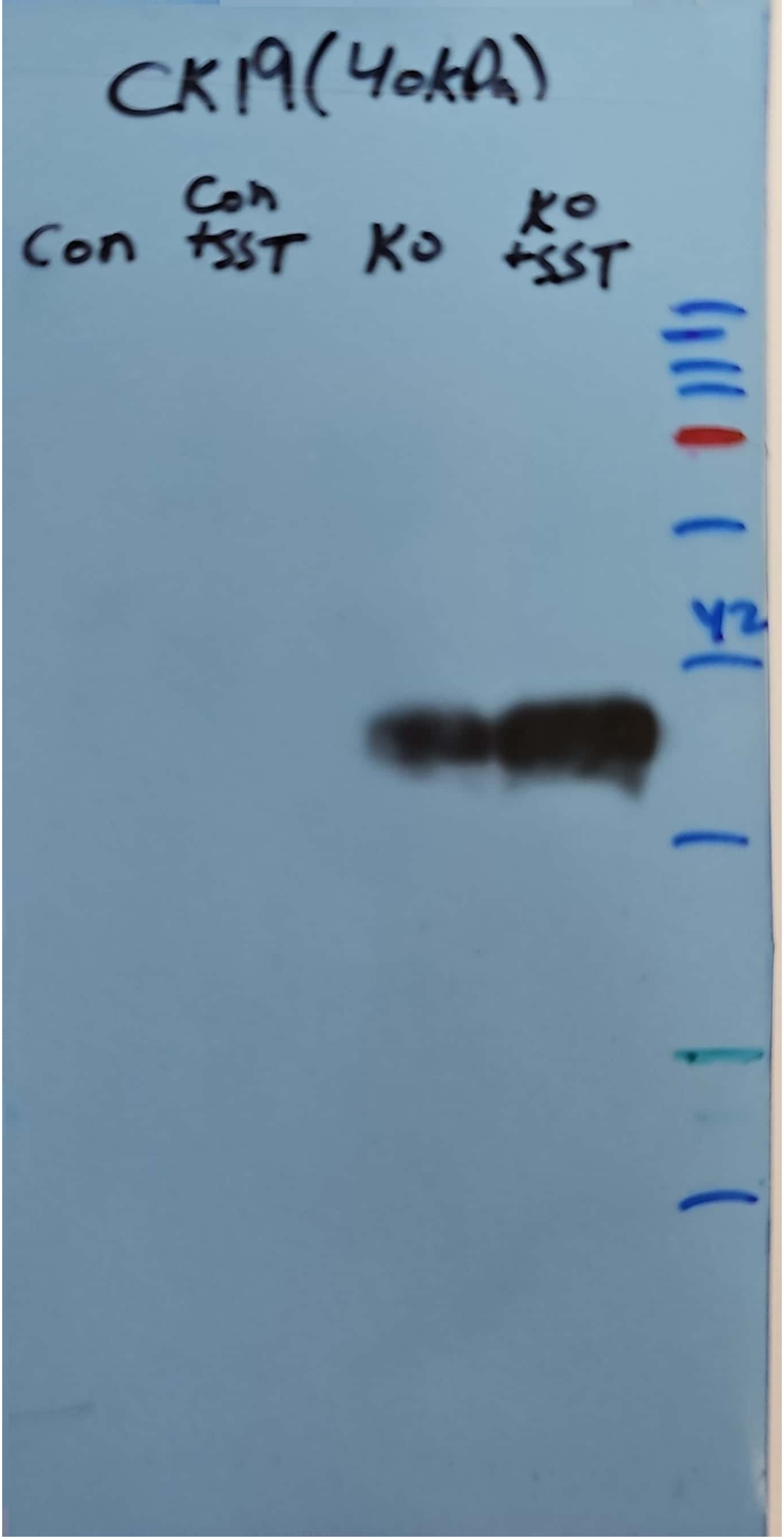

CamScanner

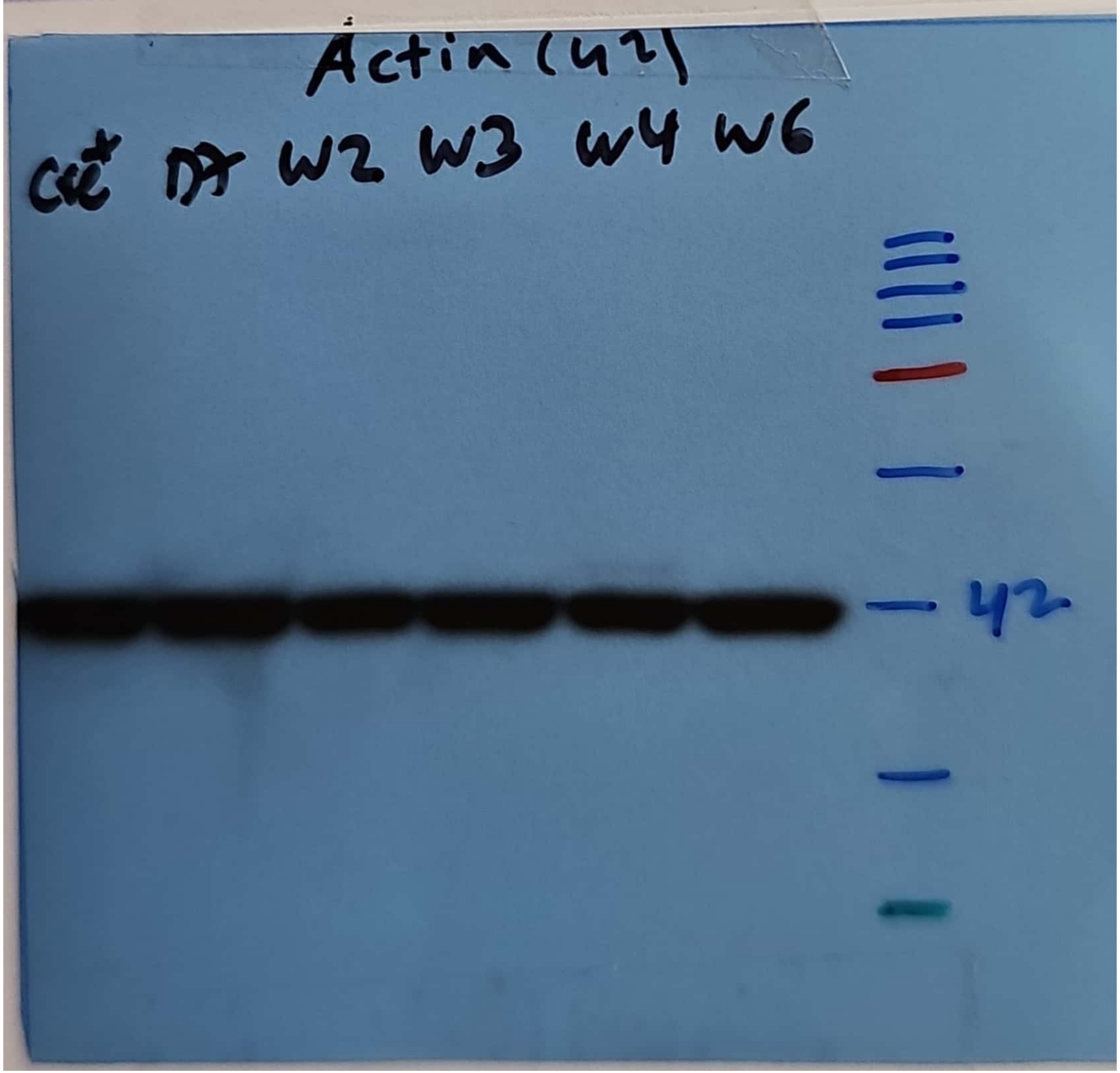

CamScanner

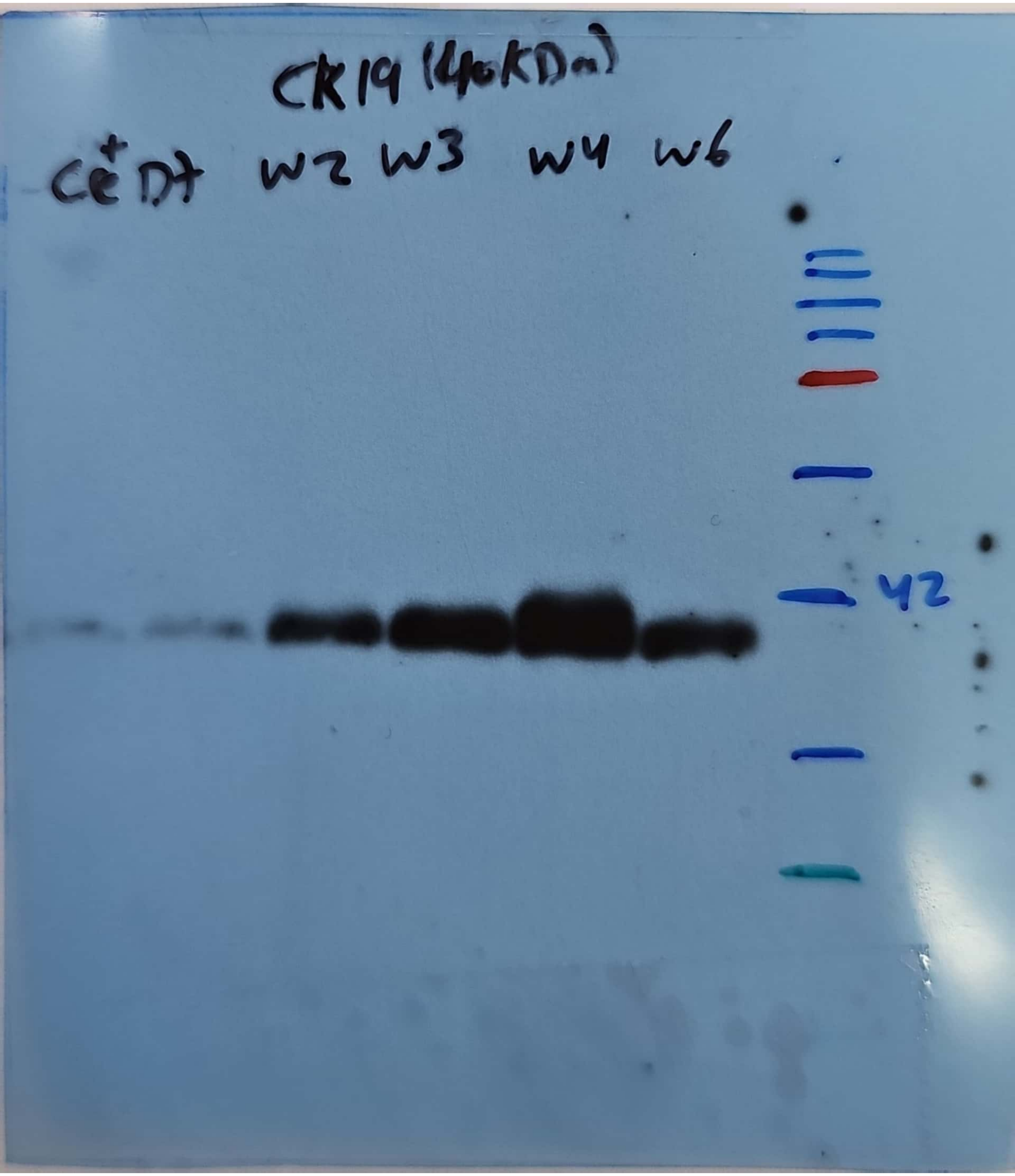

CamScanner
